# LAT1-mediated delivery of engineered R13A-MOTS-c attenuates radiation-induced lung injury via Nrf2 activation and mitochondrial protection

**DOI:** 10.1016/j.redox.2026.104204

**Published:** 2026-05-09

**Authors:** Yan-li Zhang, Guo Huang, Sheng-peng Li, Wen-long Zhang, Dan Chen, Liu-gen Jin, Qing-feng Pang, Ya-xian Wu, Jian-feng Huang

**Affiliations:** a Department of Radiation Oncology, Affiliated Hospital of Jiangnan University, 1000 Hefeng Road, Wuxi, Jiangsu province, 214000, PR China; b Wuxi School of Medicine, Jiangnan University, 1800 Lihu Avenue, Wuxi, Jiangsu province, 214122, PR China; c Department of Clinical Laboratory, The Affiliated Suzhou Hospital of Nanjing Medical University, Suzhou Municipal Hospital, Gusu School, Nanjing Medical University, 242 Guangji Road, Suzhou, Jiangsu Province, 215008, PR China; d Department of Neurosurgery, Lianshui County People's Hospital, 6 Hongri Road, Jiangsu Province, 223400, PR China; e Department of Respiratory and Critical Care Medicine, Jiangnan University Medical Center, 68 Zhongshan Road, Wuxi, Jiangsu Province, 2140002, PR China

**Keywords:** RILI, R13A-MOTS-c, LAT1, Mitochondria

## Abstract

MOTS-c exhibits substantial antioxidant and anti-inflammatory properties, yet its therapeutic potential is constrained by poor membrane permeability due to its high polarity. To overcome this limitation, we engineered R13A-MOTS-c by substituting the polar arginine at position 13 with alanine in the wild-type peptide (Met-Arg-Trp-Gln-Glu-Met-Gly-Tyr-Ile-Phe-Tyr-Pro-Arg-Lys-Leu-Arg). This modification increased the peptide's hydrophobicity index from −0.938 to −0.544, measurably improving its cellular uptake. Functional uptake assays, including competition with canonical LAT1 substrates (leucine, BCH) and LAT1 knockdown experiments, further confirmed that R13A-MOTS-c enters cells via LAT1-mediated transport. In vitro experiments revealed that R13A-MOTS-c suppressed inflammatory responses, oxidative damage, and mitochondrial impairment in MLE-12 cells. In vivo studies demonstrated that daily intraperitoneal administration of R13A-MOTS-c (5 mg/kg for 2 weeks) effectively mitigated radiation-induced pulmonary inflammation, oxidative stress, and mitochondrial dysfunction in C57BL/6 mice exposed to 20 Gy thoracic irradiation. Mechanistically, R13A-MOTS-c activated the Nrf2 signaling pathway, as evidenced by increased nuclear translocation of Nrf2 and upregulation of its downstream targets gene. These effects were abolished upon LAT1 inhibition, Nrf2 inhibition, or in Nrf2-knockout conditions. Collectively, these findings indicate that LAT1-mediated uptake of R13A-MOTS-c alleviates radiation-induced lung injury through Nrf2 pathway activation and mitochondrial function restoration, offering a promising therapeutic strategy for clinical applications.

## Graphical abstract

**Figure:**
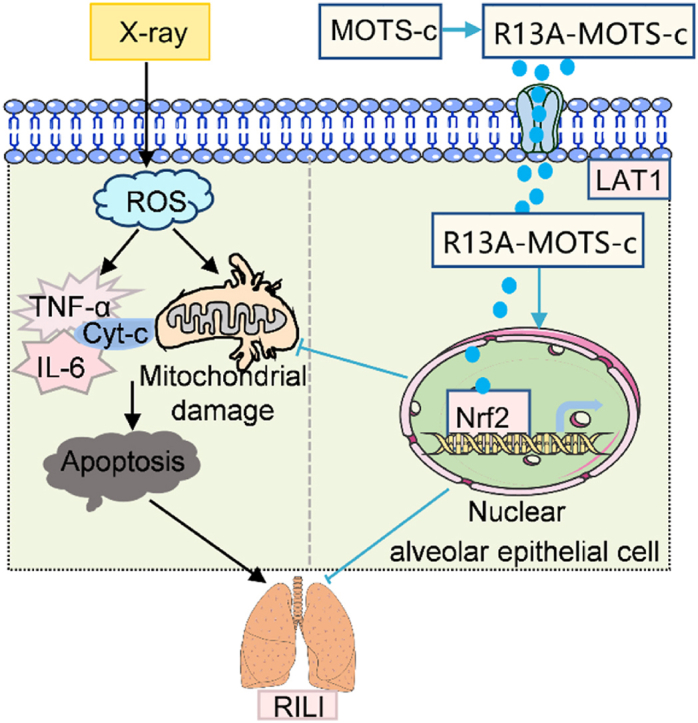

## 1 Introduction

Radiation-induced lung injury (RILI) is a common complication in patients undergoing thoracic radiotherapy for malignancies such as lung or breast cancer, significantly impairing treatment efficacy and quality of life [1]. Current clinical strategies predominantly focus on symptomatic treatments, including antioxidants, glucocorticoids, and steroidal agents. However, these interventions often yield limited therapeutic benefits and are associated with considerable side effects [2]. Alveolar epithelial cells, as the primary cellular targets in RILI, play essential roles in both injury response and tissue repair [3]. Mitochondria, which serve as the cellular energy generators and signaling centers, are central to radiation-induced damage in pulmonary epithelial cells. Mitochondria dysfunction is recognized as a key mechanism underlying RILI [4]. Specifically, radiation-induced mitochondrial damage disrupts energy metabolism, leads to excessive reactive oxygen species (ROS) accumulation, and triggers abnormal apoptotic signaling, thereby worsening RILI [3,5]. Therefore, maintaining mitochondrial function in alveolar epithelial cells represents a promising therapeutic strategy for mitigating RILI.

Mitochondrial open reading frame of the 12S rRNA type-c (MOTS-c), a 16-amino acid peptide encoded by mitochondrial DNA (mtDNA), exerts its protective effects by enhancing transcription factor activity [6,7].Specifically, under conditions of oxidative stress induced by hydrogen peroxide or high glucose, MOTS-c rapidly translocate from mitochondria to the nucleus within 30 min. In the nucleus, MOTS-c interacts with the nuclear transcription factor nuclear factor erythroid 2–related factor 2 (Nrf2) to promote the expression of key genes essential for mitochondrial biogenesis [7]. MOTS-c enhances mitochondrial homeostasis by inhibiting mitochondrial ROS production and increasing mitochondrial membrane potential [8,9]. Recent studies further highlight its protective effects against obesity, diabetes, osteoporosis, and aging through the improvement of mitochondrial function [[10], [11], [12], [13]]. However, the therapeutic potential of MOTS-c is limited by its poor cell membrane permeability [14,15]. To address this challenge, various delivery systems, such as inorganic nanoparticles, liposomes, nanogels, polymeric nanoparticles, and protein-based carriers, have been developed to facilitate intracellular peptide delivery [16]. Despite these advancements, carrier-mediated systems often experience lysosomal degradation post-endocytosis, thereby compromising therapeutic efficacy. Recent advances demonstrate that chemical modification of peptide amino acid residues to enhance specific binding with membrane amino acid transporters enables direct intracellular peptide delivery [16,17]. Multiple amino acid transporters on the cell membrane mediate transporter-assisted, non-endocytic amino acid influx to maintain cellular metabolic homeostasis. Therefore, identifying membrane proteins capable of efficiently achieving transmembrane delivery of peptide substances holds significant promise to overcome the clinical translation challenges of MOTS-c as a therapeutic peptide.

The L-type amino acid transporter 1 (LAT1) which is highly expressed in pulmonary epithelial cells, plays a pivotal role in cellular physiology and drug delivery [18,19]. As a Na^+^-independent and pH-independent transmembrane transporter, LAT1 efficiently recognizes and transports various amino acids, including nonpolar aliphatic amino acids (leucine, isoleucine, and valine), aromatic amino acids (phenylalanine, tyrosine, and tryptophan), basic amino acids (histidine), and polar neutral amino acids (methionine). Additionally, LAT1 demonstrates remarkable capability in transporting exogenous peptides [[20], [21], [22]]. Beyond its canonical role in amino acid transport, LAT1 facilitates the cellular uptake of thyroid hormones, including triiodothyronine (T3) and thyroxine (T4) [23]. Notably, LAT1 mediates the blood-brain barrier penetration of various neuroactive drugs, such as the antiepileptics gabapentin and pregabalin, as well as the anti-Parkinsonian agent l-DOPA [[24], [25], [26]]. Although no studies have demonstrated LAT1-mediated MOTS-c transport, considering LAT1's specific recognition capability for neutral hydrophobic peptides and its successful applications in drug delivery, targeting LAT1 could potentially represent a promising strategy to overcome the cell membrane penetration barrier of MOTS-c.

In this study, we designed and synthesized a novel R13A-MOTS-c peptide by substituting arginine at position 13 with alanine. Cellular immunofluorescence assays demonstrated that R13A-MOTS-c exhibited significantly enhanced cell membrane penetration compared to wild-type MOTS-c. In a C57BL/6 mouse model subjected to 20 Gy irradiation followed by 5 mg/kg R13A-MOTS-c intraperitoneal injection for two weeks, R13A-MOTS-c markedly attenuated inflammatory responses, oxidative stress, alveolar epithelial apoptosis, and mitochondrial damage. Experiments using MLE-12 cells further confirmed R13A-MOTS-c's efficacy in mitigating radiation-induced oxidative stress and mitochondrial dysfunction. Mechanistic studies showed that R13A-MOTS-c's enhanced LAT1 affinity facilitates nuclear entry and activates the Nrf2 transcription factor. These findings indicate that R13A-MOTS-c is a promising candidate for RILI prevention and treatment, offering novel therapeutic strategies for radiation-induced lung injury management.

## 2 Results

### 2.1 Decreased levels of MOTS-c and elevated oxidative stress were observed in peripheral blood of RILI patients

**Fig. 1:**
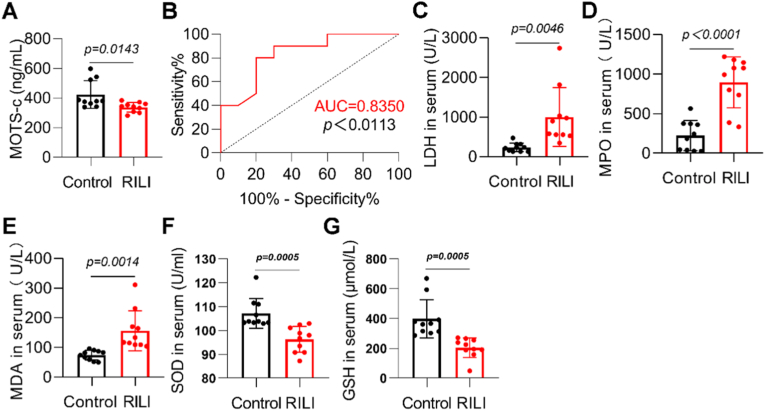
Decreased levels of MOTS-c and elevated oxidative stress were observed in peripheral blood of RILI patients. The control group (Control) comprised thoracic cancer patients who did not develop RILI after radiotherapy, and the RILI group (RILI) consisted of thoracic cancer patients who developed RILI after radiotherapy. A) Serum MOTS-c protein levels were quantified by ELISA. B) ROC curve analysis of MOTS-c protein concentrations. C-G) Serum biomarkers (LDH, MPO, MDA, SOD, and GSH) were measured using commercial assay kits. Data are presented as mean ± SD (*n* = 10). Student's *t*-test were used for statistical analysis.

To investigate the changes of MOTS-c in patients with RILI, we collected serum samples from 10 thoracic cancer patients who developed RILI after radiotherapy (RILI) and 10 thoracic cancer patients who did not develop RILI after radiotherapy as controls. The basic characteristics of the patients were shown in Table S1. ELISA results revealed that the MOTS-c content in the serum of RILI patients was significantly lower than that in the control group (Fig. 1A). Furthermore, ROC curve analysis revealed an AUC value of 0.8350 for MOTS-c in RILI patients, suggesting its potential diagnostic and prognostic significance for RILI (Fig. 1B). Additionally, serum levels of lactate dehydrogenase (LDH), myeloperoxidase (MPO), and malondialdehyde (MDA) were significantly elevated in RILI patients (Fig. 1C–E), whereas levels of superoxide dismutase (SOD) and glutathione (GSH) were decreased (Fig. 1F and G). These findings suggest that the decreased levels of MOTS-c in the serum of RILI patients could serve as a potential diagnostic marker for RILI.

### 2.2 Engineered R13A-MOTS-c demonstrated enhanced membrane penetration capability

**Fig. 2:**
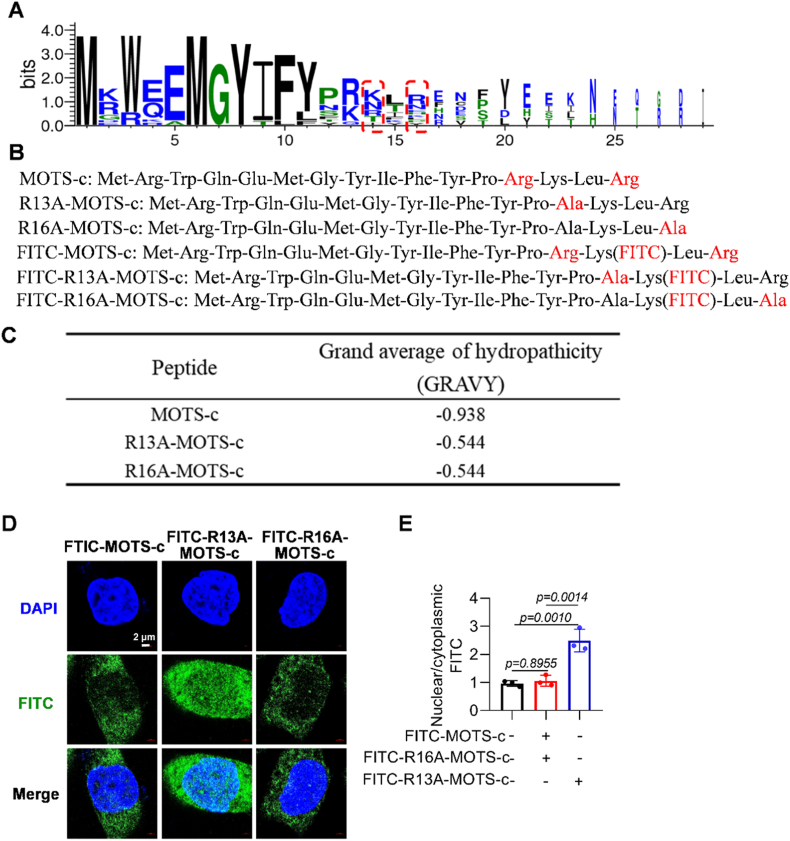
Engineered R13A-MOTS-c demonstrated enhanced membrane penetration capability. A) Conservation analysis of MOTS-c peptide residues across 14 species. B) Sequences of MOTS-c and its derivatives. C) Comparative hydrophobicity coefficients of three peptide variants. D, E) Nuclear localization of three MOTS-c variants assessed by immunofluorescence (scale bar: 2 μm). Data were presented as mean ± SD (*n* = 3). One-way analysis of variance (ANOVA) followed by Tukey's post hoc test were used for statistical analysis.

The first 11 amino acid residues of the wild-type MOTS-c peptide sequence (Met-Arg-Trp-Gln-Gln-Met-Gly-Tyr-Ile-Phe-Tyr-Pro-Arg-Lys-Leu-Arg) were highly conserved across different species (Fig. 2A). After replacing the arginine residues at positions 13 and 16 of the wild-type MOTS-c peptide with alanine, the resulting R13A-MOTS-c and R16A-MOTS-c peptides exhibited an increased average hydrophobicity index from −0.938 to −0.544 (Fig. 2B and C), indicating a significantly enhanced membrane-penetrating capacity. However, subsequent cellular immunofluorescence assays unexpectedly revealed no improvement in membrane permeability for R16A-MOTS-c (Fig. 2D and E). Consequently, this study focused specifically on R13A-MOTS-c to investigate its potential protective effects against RILI.

### 2.3 Membrane LAT1 mediated the intracellular transport of R13A-MOTS-c

**Fig. 3:**
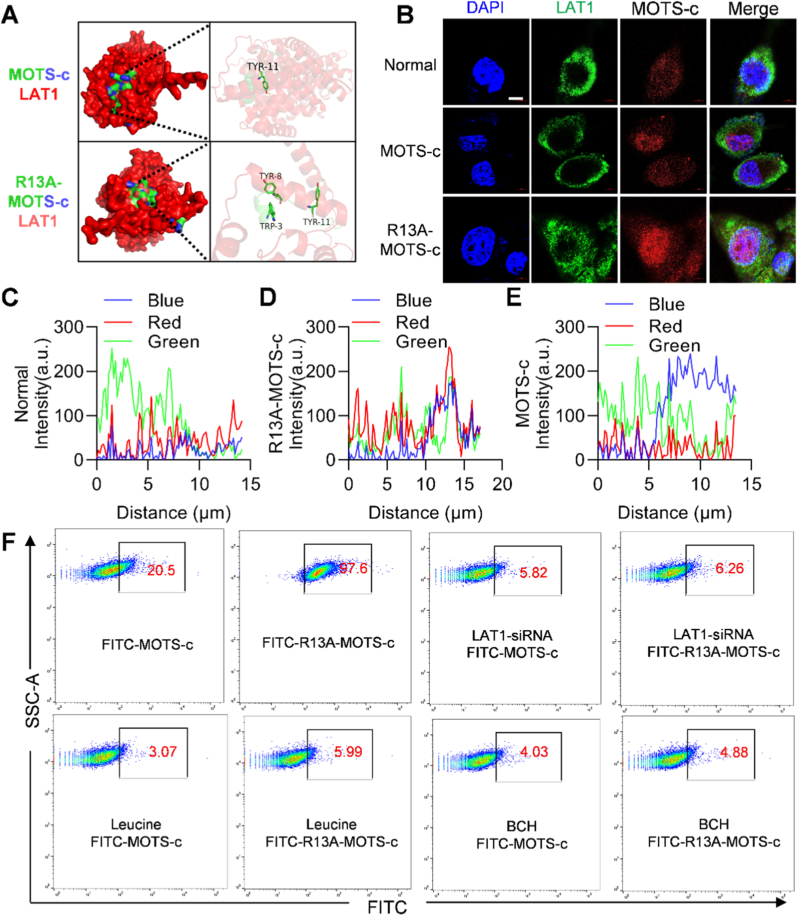
Membrane LAT1 mediated the intracellular transport of R13A-MOTS-c. A) The binding ability of MOTS-c and MOTS-cR13A to LAT1 was observed by molecular docking. B-E) Immunofluorescence staining of cells with LAT1 (green), DAPI (blue), and MOTS-c (red) (scale bar: 2 μm). F) The uptake of FITC-MOTS-c and FITC-R13A-MOTS-c in MLE-12 cells was detected by flow cytometry. Data were presented as mean ± SD (*n* = 3).

LAT1 membrane protein is highly abundant in pulmonary vascular epithelial cells and has a strong ability to specifically recognize and transport peptides into cells. To further explore the mechanism of the enhanced permeability of R13A-MOTS-c, relevant experiments were performed. The results of molecular docking experiments showed that R13A-MOTS-c had a stronger affinity for membrane protein LAT1 than wild-type MOTS-c (Fig. 3A). Immunofluorescence results showed that R13A-MOTS-c had enhanced colocalization of membrane protein LAT1 compared with wild-type MOTS-c, resulting in increased intracellular content of R13A-MOTS-c (Fig. 3B–E). We have quantitatively assessed the cellular uptake of the peptide using flow cytometry and supplemented the study with competition assays using canonical LAT1 substrates (leucine and BCH). The results showed that the uptake of R13A-MOTS-c was significantly inhibited by both leucine and BCH, further confirming that the entry of this peptide is dependent on the LAT1 transport pathway (Fig. 3F and Supplementary Fig. S1). To further validate LAT1's role, we employed siRNA-mediated knockdown and plasmid-based overexpression (Supplementary Fig. S2A–D). Co-IP experiments indicated a stronger binding affinity between R13A-MOTS-c and the LAT1 protein relative to wild-type MOTS-c (Supplementary Fig. S2E). Confocal imaging confirmed that LAT1 siRNA markedly diminished the cellular uptake of R13A-MOTS-c, whereas LAT1 reconstitution via overexpression plasmids successfully rescued this uptake (Supplementary Fig. S2F and G). The above results indicated that R13A-MOTS-c binds to the LAT1 protein, thereby promoting its transport into the cells.

### 2.4 R13A-MOTS-c alleviated radiation-induced inflammatory response and oxidative stress damage in MLE-12 cells

**Fig. 4:**
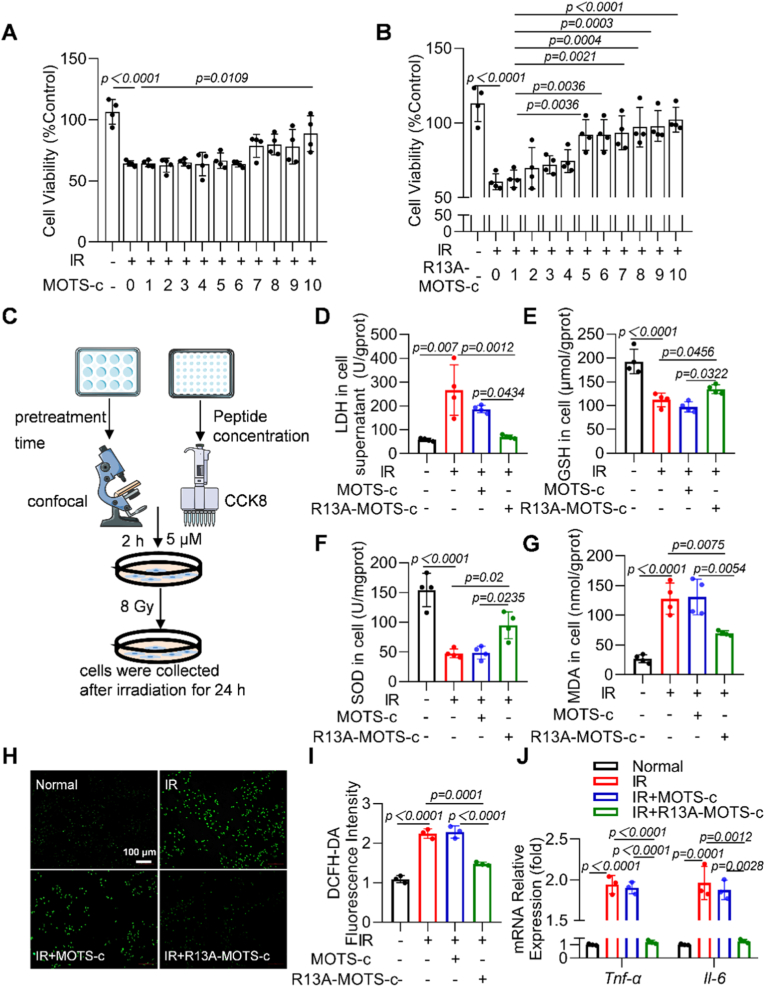
R13A-MOTS-c alleviated radiation-induced inflammatory response and oxidative stress damage in MLE-12 cells. A, B) The effects of MOTS-c and R13A-MOTS-c on MLE-12 cells were detected by CCK-8 assay. C) Irradiation scheme for MLE-12 cells. D-G) LDH, GSH, SOD and MDA levels in MLE-12 cells. H, I) Intracellular ROS levels were measured using the DCFH-DA fluorescent probe (scale bar: 100 μm). J) The mRNA levels of *Il-6* and *Tnf-α* were determined by RT-qPCR. Data were presented as mean ± SD (*n* = 3). One-way analysis of variance (ANOVA) followed by Tukey's post hoc test were used for statistical analysis.

R13A-MOTS-c exerted its function by entering the nucleus and activating transcription factors. To determine the optimal time point for its nuclear translocation, we performed intracellular tracing using FITC-labeled R13A-MOTS-c. Confocal microscopy results revealed that the accumulation of FITC-R13A-MOTS-c in the nucleus reached its peak after a 2-h pretreatment period (Supplementary Fig. S3A and B). Based on this finding, a 2 h pretreatment with FITC-R13A-MOTS-c was adopted for all subsequent experiments. Meanwhile, we evaluated the optimal working concentration of R13A-MOTS-c via CCK-8 assays. As shown in Fig. 4A and B, 5 μM R13A-MOTS-c effectively alleviated radiation-induced injury in MLE-12 cells, whereas a higher concentration of 10 μM wild-type MOTS-c was required to achieve a comparable protective effect, indicating that R13A-MOTS-c possesses superior cytoprotective efficacy. Based on these findings, subsequent *in vitro* experiments employed a 2 h pretreatment with 5 μM R13A-MOTS-c to further investigate its role in mitigating radiation-induced damage (Fig. 4C). The experimental results demonstrated that R13A-MOTS-c intervention reduced the levels of LDH in the supernatant of radiation-exposed MLE-12 cells (Fig. 4D). Additionally, we evaluated oxidative stress-related markers and found that R13A-MOTS-c treatment alleviated the radiation-induced decrease in GSH and SOD levels while suppressing the increase in MDA levels (Fig. 4E–G). Moreover, R13A-MOTS-c inhibited the radiation-induced elevation of ROS levels (Fig. 4F–I). The mRNA expression levels of *Tnf-α* and *Il-6* were reduced in R13A-MOTS-c-treated MLE-12 cells (Fig. 4J). Taken together, these results indicate that R13A-MOTS-c holds significant potential for mitigating radiation-induced oxidative stress and inflammatory responses.

### 2.5 R13A-MOTS-c relieved radiation-induced inflammatory response and oxidative stress damage in RILI mice

**Fig. 5:**
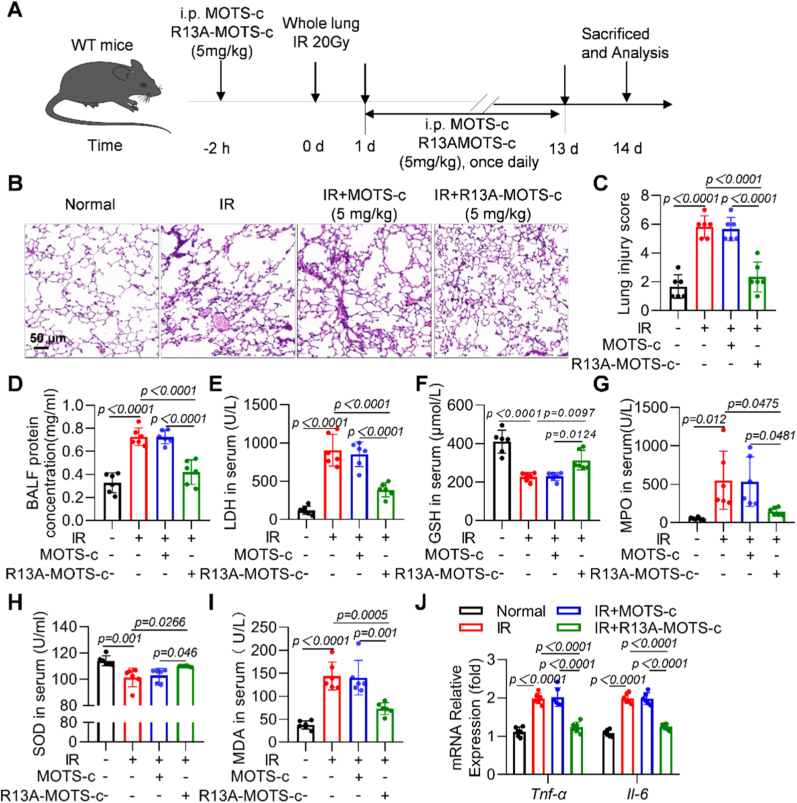
R13A-MOTS-c relieved radiation-induced inflammatory response and oxidative stress damage in RILI mice. A) Schematic timeline of R13A-MOTS-c and MOTS-c intervention for radiation-induced RP in WT mice. B, C) Lung tissue sections were stained with hematoxylin-eosin (HE), scale bar = 50 μm. D) Total protein concentration in the BALF were detected. E-H) LDH, GSH, MPO, SOD and MDA levels in mice serum were determined. J) The mRNA levels of *Il-6* and *Tnf-α* were determined by RT-qPCR in lung tissue. Data were presented as mean ± SD (*n* = 5). One-way analysis of variance (ANOVA) followed by Tukey's post hoc test were used for statistical analysis.

To further investigate the potential therapeutic effects of R13A-MOTS-c in RILI, mice were exposed to radiation and subsequently treated with either 5 mg/kg of wild-type MOTS-c or the R13A-MOTS-c (Fig. 5A). Western blot results demonstrated that radiation induced a reduction in MOTS-c protein levels in mouse lung tissues (Supplementary Fig. S4A and B). The H&E staining results of lung tissue showed that mice exposed to radiation alone exhibited significant morphological damage, including edema, disruption of alveolar structures, and extensive infiltration of inflammatory cells into the lung parenchyma. In contrast, these damages were markedly alleviated in mice treated with R13A-MOTS-c. Notably, wild-type MOTS-c at the same dose did not demonstrate any mitigating effects on RILI (Fig. 5B and C). Further analysis revealed that R13A-MOTS-c treatment reduced the protein concentration (Fig. 5D) and LDH levels (Fig. 5E) in BALF, indicating reduced lung tissue damage. The therapeutic effect of wild-type MOTS-c at 10 mg/kg on mice with RP was also evaluated in this study. HE staining results demonstrated that MOTS-c at this dosage effectively alleviated radiation-induced lung tissue damage (Supplementary Fig. S4C and D), which was consistent with our previous study [4]. These findings indicate that while wild-type MOTS-c exhibited protective effects at 10 mg/kg, R13A-MOTS-c demonstrated efficacy at a lower dose of 5 mg/kg, suggesting an improved dose–response profile. To comprehensively assess the oxidative stress status, biochemical indicators in the serum were measured, including GSH, MPO, SOD, and MDA. The results showed that R13A-MOTS-c treatment decreased the levels of MPO and MDA while significantly increasing the levels of SOD and GSH (Fig. 5F–I), collectively indicating an improvement in oxidative stress. Additionally, the mRNA expression levels of the inflammatory cytokines *Il-6* and *Tnf-α* in lung tissue was examined. The results demonstrated that the expression levels of both cytokines were reduced following R13A-MOTS-c treatment (Fig. 5J). In summary, these experimental results suggest that R13A-MOTS-c effectively alleviated oxidative damage, inflammatory responses, and morphological lung injury in RILI mice.

### 2.6 R13A-MOTS-c reduced radiation-induced mitochondrial damage in MLE-12 cells

**Fig. 6:**
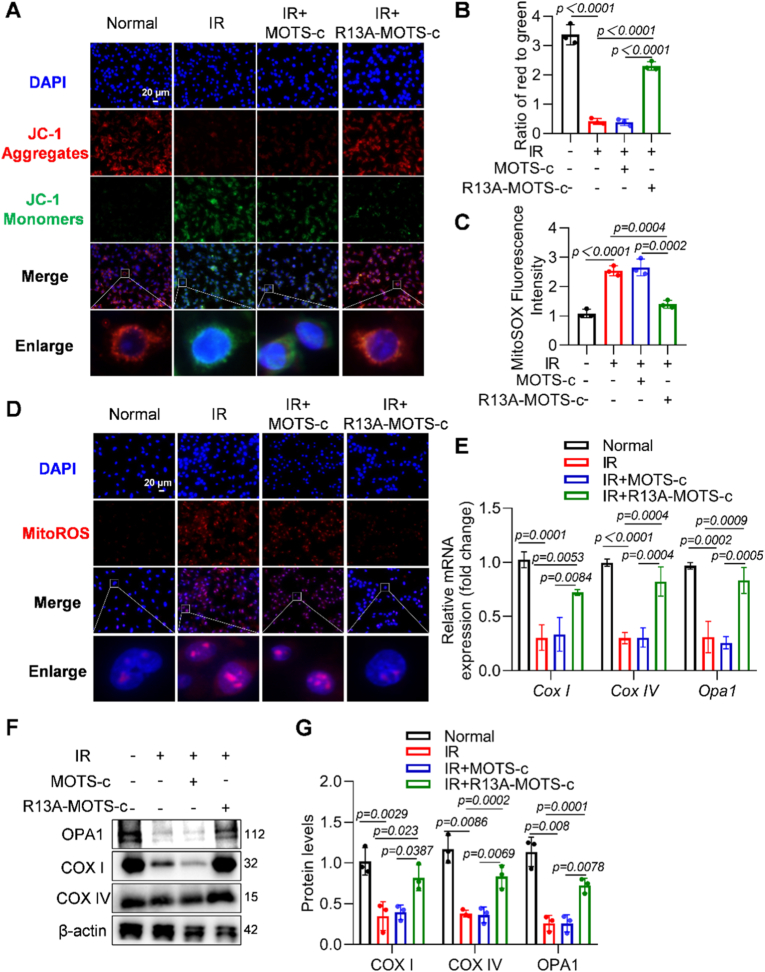
R13A-MOTS-creduced radiation-induced mitochondrial damage in MLE-12 cells. A, B) Mitochondrial membrane potential in MLE-12 cells was measured using the JC-1 staining kit (scale bar: 20 μm). C, D) The mtROS levels in MLE-12 cells were detected using the MitoSOX Red fluorescent probe (scale bar: 20 μm). E) The mRNA expression levels of *Cox IV*, and *Opa1* were determined by RT-qPCR. F, G) Protein levels of COX I, COX IV, and OPA1 in MLE-12 cells were analyzed by Western blot. Data were presented as mean ± SD (*n* = 3). One-way analysis of variance (ANOVA) followed by Tukey's post hoc test were used for statistical analysis.

To investigate the protective effects of R13A-MOTS-c on radiation-induced mitochondrial damage, we conducted a series of experiments assessing mitochondrial function. The results showed that R13A-MOTS-c effectively alleviated the transition of the JC-1 fluorescent probe from aggregates (red) to monomers (green), thereby maintaining the stability of the mitochondrial membrane potential (Fig. 6A and B). Cellular immunofluorescence results revealed that, compared to the radiation group, supplementation with R13A-MOTS-c markedly reduced the red fluorescence signals of MitoSOX and Rhod-2AM in MLE-12 cell (Fig. 6C, D and Supplementary Fig. S5A and B). This indicates that R13A-MOTS-c could reduce mitochondrial ROS production and potentially regulate calcium homeostasis. Moreover, R13A-MOTS-c up-regulated the mRNA expression of *Cox I*, *Cox IV*, and *Opa1* in MLE-12 cells (Fig. 6E) and increased the protein levels of COXI, COXIV, and OPA1 (Fig. 6F and G). These findings collectively suggest that R13A-MOTS-c promotes mitochondrial structural and functional integrity in MLE-12 cells.

### 2.7 R13A-MOTS-c mitigated radiation-induced apoptosis in MLE-12 cells

**Fig. 7:**
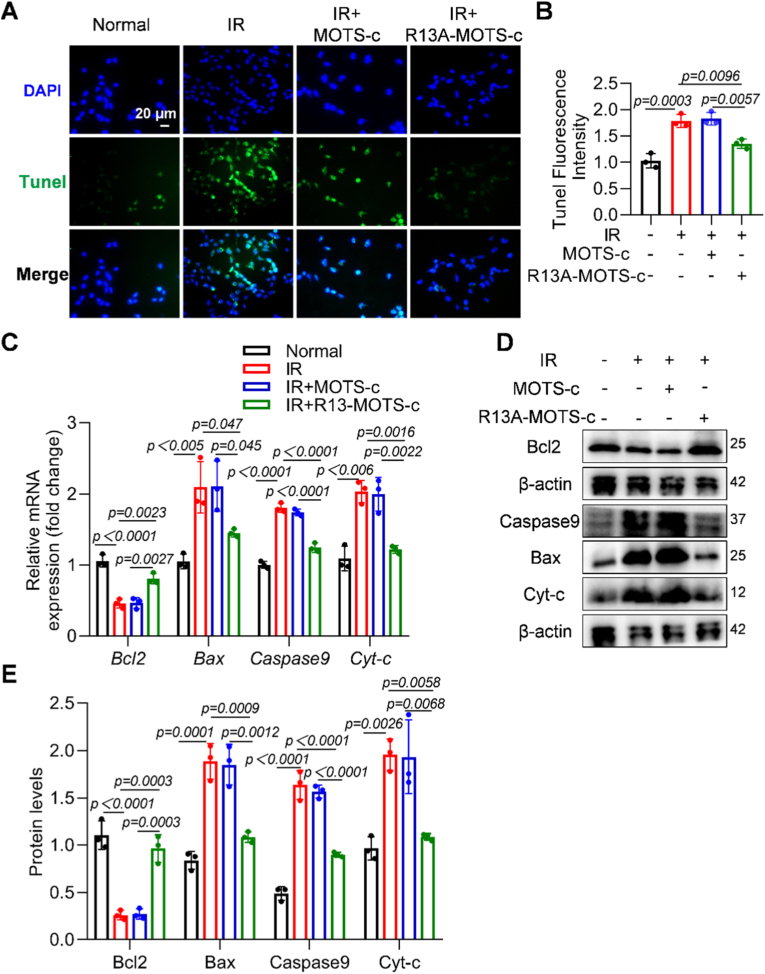
R13A-MOTS-c mitigated radiation-induced apoptosis in MLE-12 cells. A, B) Apoptosis levels in MLE-12 cells were detected by TUNEL staining (scale bar: 20 μm). C) The mRNA expression levels of *Bcl2*, *Bax*, *Caspase9* and *Cyt-c* in MLE-12 cells were measured by RT-qPCR. D, E) Protein levels of Bcl2, Bax, Caspase9 and Cyt-c in MLE-12 cells were analyzed by Western blot. Data were presented as mean ± SD (*n* = 3). One-way analysis of variance (ANOVA) followed by Tukey's post hoc test were used for statistical analysis.

TUNEL staining results demonstrated that R13A-MOTS-c effectively alleviated radiation-induced cell apoptosis (Fig. 7A and B). Further studies revealed that R13A-MOTS-c significantly down-regulated the mRNA expression of pro-apoptotic genes *Bax*, *Caspase9*, and *Cyt-c*, while up-regulating the expression of the anti-apoptotic gene *Bcl2* (Fig. 7C). Additionally, R13A-MOTS-c reduced the protein levels of Bax, Caspase9, and Cyt-c, while increasing the protein level of Bcl2 (Fig. 7D and E). Collectively, these findings suggest that R13A-MOTS-c mitigates radiation-induced cell apoptosis by regulating the expression of apoptosis-related genes at both transcriptional and translational levels.

### 2.8 R13A-MOTS-c suppressed radiation-induced apoptosis and mitochondrial damage in RILI mice

**Fig. 8:**
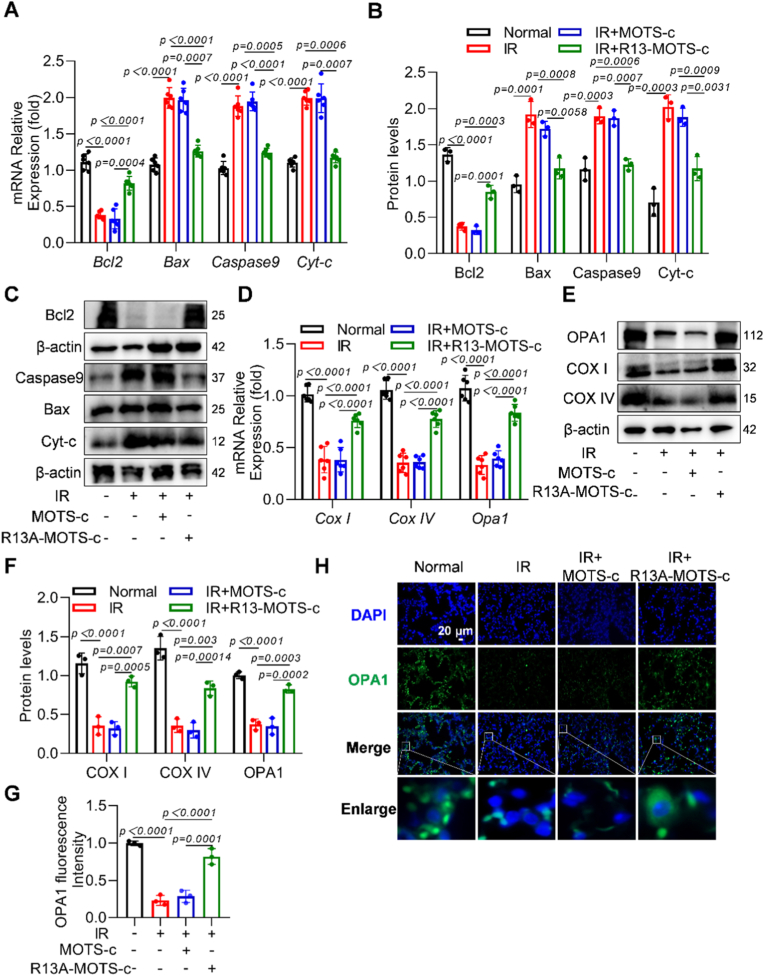
R13A-MOTS-c suppressed radiation-induced apoptosis and mitochondrial damage in RILI mice. A) The mRNA expression levels of *Bcl2*, *Bax*, *Caspase9* and *Cyt-c* in lung tissues were measured by RT-qPCR. B, C) Protein levels of Bcl2, Bax, Caspase9 and Cyt-c in lung tissues were analyzed by Western blot. D) The mRNA levels of *Opa1*, *Cox I* and *Cox IV* in lung tissues were determined by RT-qPCR. E, F) Protein content of OPA1, COX I and COX IV was detected by Western blot. G, H) Immunofluorescence analysis of lung tissues stained with OPA1 (green) and DAPI (blue) (scale bar: 20 μm). Data were presented as mean ± SD (*n* = 5). One-way analysis of variance (ANOVA) followed by Tukey's post hoc test were used for statistical analysis.

To further validate the protective effects of R13A-MOTS-c in RILI mice, we examined changes in apoptosis-related genes and mitochondrial function-related indicators in lung tissue. The experimental results showed that R13A-MOTS-c down-regulated the mRNA expression of pro-apoptotic genes *Bax*, *Caspase9*, and *Cyt-c*, while up-regulating the expression of the anti-apoptotic gene *Bcl2* (Fig. 8A). Consistent with these findings, protein levels exhibited similar trends (Fig. 8B and C). Furthermore, after R13A-MOTS-c treatment, the mRNA expression levels of mitochondrial function-related genes *Cox I*, *Cox IV*, and *Opa1* in lung tissue were significantly increased (Fig. 8D), and the corresponding protein levels also showed consistency with the gene expression data (Fig. 8E and F). Immunofluorescence staining of lung tissue further demonstrated that R13A-MOTS-c reversed the radiation-induced reduction in OPA1 and TFAM protein levels (Fig. 8G, H and Supplementary Fig. S5C and D). In summary, these findings suggest that R13A-MOTS-c provides significant protection against RILI by inhibiting apoptosis and enhancing mitochondrial function.

### 2.9 Engineered R13A-MOTS-c exerted radioprotection through an Nrf2-dependent mechanism

**Fig. 9:**
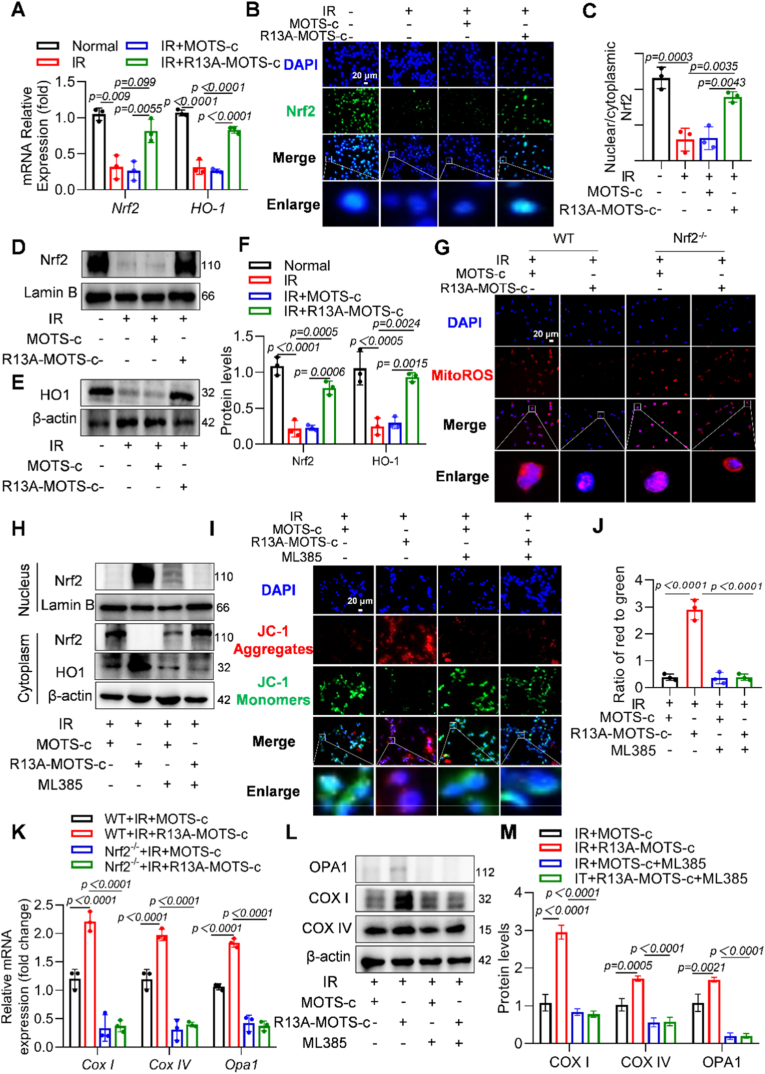
Engineered R13A-MOTS-c exerted radioprotection through an Nrf2-dependent mechanism. A) The mRNA expression levels of *Nrf2* and *HO-1* in MLE-12 cells were measured by RT-qPCR. B, C) Immunofluorescence analysis of MLE-12 stained with Nrf2 (green) and DAPI (blue) (scale bar: 20). D-F) Protein levels of Nrf2 and HO-1 were analyzed by Western blot. G) The mtROS levels in primary lung epithelial cells were detected using the MitoSOX Red fluorescent probe (scale bar: 20 μm), H) Protein levels of Nrf2 and HO-1 were analyzed by Western blot. I, J) Mitochondrial membrane potential in MLE-12 cells was measured using the JC-1 staining kit (scale bar: 20 μm). K) The mRNA expression levels of *Cox I*, *Cox IV* and *Opa1* were determined by RT-qPCR in primary lung epithelial cells. L, M) Protein levels of COX I, COX IV, and OPA1 in MLE-12 cells were analyzed by Western blot. Data were presented as mean ± SD (*n* = 3). One-way analysis of variance (ANOVA) followed by Tukey's post hoc test were used for statistical analysis.

The R13A-MOTS-c peptide exerts cytoprotective effects primarily through the activation of transcription factors. To validate the intracellular bioactivity of this modified peptide, we assessed the levels of the transcription factor Nrf2 and its downstream genes. RT-qPCR analysis revealed that irradiation induced a decrease in the mRNA levels of both Nrf2 and its downstream gene HO-1. Treatment with R13A-MOTS-c effectively mitigated this reduction, significantly elevating the transcript levels of Nrf2 and HO-1(Fig. 9A). Cellular immunofluorescence demonstrated that R13A-MOTS-c promoted the nuclear translocation of the Nrf2 protein (Fig. 9B and C). Western blot analysis of isolated nuclear protein extracts further demonstrated that irradiation led to reduced Nrf2 protein levels within the nucleus, an effect that was markedly reversed by R13A-MOTS-c (Fig. 9D–F). Consistently, R13A-MOTS-c also counteracted the irradiation-induced downregulation of HO-1 protein expression (Fig. 9E and F). Collectively, these results indicated that R13A-MOTS-c activates the Nrf2/HO-1 signaling pathway. To further investigate whether the cytoprotective effect of R13A-MOTS-c depends on the Nrf2 pathway, we isolated primary pulmonary epithelial cells from wild-type (WT) mice and Nrf2-knockout (Nrf2^−/−^) mice (Supplementary Fig. S6A). The results showed that in the absence of Nrf2, R13A-MOTS-c failed to effectively mitigate irradiation-induced elevation of mitochondrial reactive oxygen species (Fig. 9G). Subsequently, we used the specific Nrf2 inhibitor ML385 to suppress Nrf2 protein expression and performed further functional assays (Supplementary Fig. S6B). Western Blot results showed that R13A-MOTS-c promoted the nuclear translocation of Nrf2 and the activation of its downstream pathway, while ML385 inhibited this effect (Fig. 9H). The results indicated that under Nrf2 inhibition, R13A-MOTS-c likewise could not effectively alleviate irradiation-induced increases in mitochondrial ROS (Supplementary Fig. S6C and D) or the loss of mitochondrial membrane potential Fig. 9I and J). In addition, we extracted mRNA from primary alveolar epithelial cells. The results demonstrated that in Nrf2-knockout conditions, R13A-MOTS-c treatment also failed to prevent radiation-induced reduction in the mRNA levels of mitochondrial-related genes (*CoxI*, *CoxIV*, and *Opa1*) (Fig. 9K). In the MLE-12 cell line, similar observations were made: under Nrf2 inhibition, R13A-MOTS-c could not prevent the decreased expression of mitochondrial-related genes (*CoxI, CoxIV,* and *Opa1*) at both the mRNA (Supplementary Fig. S6E) and protein levels (Fig. 9L and M). In conclusion, R13A-MOTS-c exerted its radioprotective effects through an Nrf2-dependent mechanism.

### 2.10 Inhibition of LAT1 abolished the protective of R13A-MOTS-c against mitochondrial damage in MLE-12 cells

**Fig. 10:**
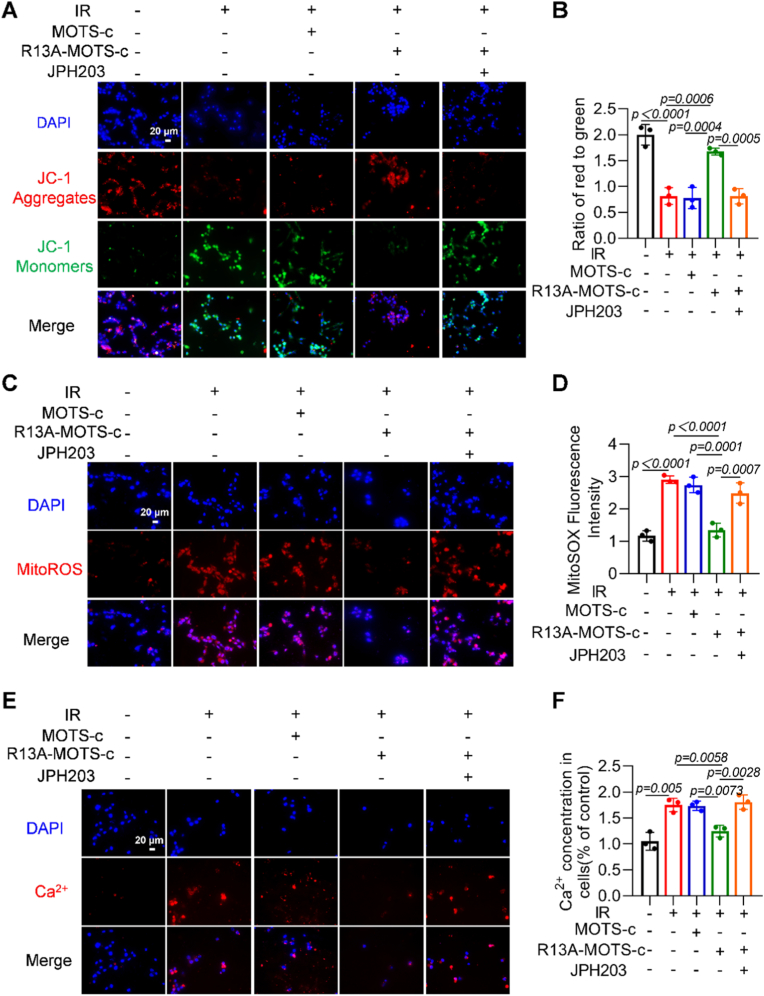
Inhibition of LAT1 abolished the protective of R13A-MOTS-c against mitochondrial damage in MLE-12 cells. A, B) Mitochondrial membrane potential in MLE-12 cells was assessed using JC-1 staining kit (scale bar: 20 μm). C, D) mtROS levels in MLE-12 cells were detected using MitoSOX™ Red fluorescent probe (scale bar: 20 μm). E, F) Rhod-2 AM Red fluorescent probe was used to detect calcium content in MLE-12 (scale bar: 20 μm). Data were presented as mean ± SD (*n* = 5). One-way analysis of variance (ANOVA) followed by Tukey's post hoc test were used for statistical analysis.

To further validate whether R13A-MOTS-c exerts its protective effects through LAT1-mediated transport, LAT1 levels were examined in irradiated MLE-12 cells (Supplementary Fig. S7A and B) and lung tissues from RP mice (Supplementary Fig. S7C–E). The results showed that radiation did not induce significant changes in LAT1 mRNA level and protein content. Subsequently, we used the LAT1-specific inhibitor JPH203 to investigate the underlying mechanism. To rule out potential off-target effects of JPH203 at a concentration of 25 μM, we performed Co-IP experiments. The results showed that the presence of MOTS-c and R13A-MOTS-c did not interfere with the inhibitory effect of JPH203 on LAT1 (Supplementary Fig. S7F), indicating that JPH203 exhibited no detectable off-target effects under these conditions. The results of apoptosis- and mitochondrial function-related indicators showed that neither JPH203 treatment alone nor its combination with MOTS-c had a significant effect on these parameters (Supplementary Fig. S6G and H). Based on these findings, the JPH203-alone group and the MOTS-c + JPH203 co-treatment group were omitted in subsequent experiments. Using the JC-1 fluorescent probe to evaluate mitochondrial membrane potential, we found that R13A-MOTS-c maintained the stability of the mitochondrial membrane potential, as evidenced by a reduced transition of the fluorescent probe from aggregates (red) to monomers (green). This protective effect was eliminated after pretreatment with JPH203 (Fig. 10A and B). Additionally, following JPH203 treatment, R13A-MOTS-c did not reduce MitoSOX fluorescence intensity and calcium levels (Fig. 10C–F). These results indicate that R13A-MOTS-c effectively prevents mitochondrial dysfunction through a LAT1-dependent mechanism, and inhibition of LAT1 function abolished the protective effects of R13A-MOTS-c.

### 2.11 Inhibition of LAT1 diminished the protective effect of R13A-MOTS-c against apoptosis and inflammatory response in MLE-12 cells

**Fig. 11:**
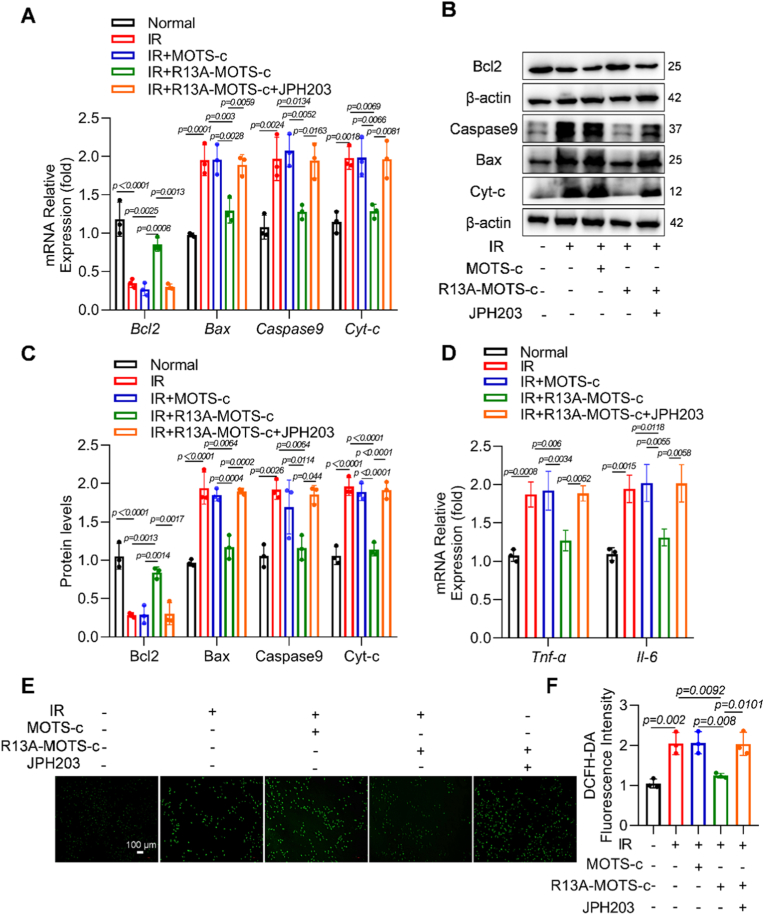
Inhibition of LAT1 attenuated the protective effect of R13A-MOTS-c against apoptosis and inflammatory response in MLE-12 cells. A) The mRNA expression levels of *Bcl2*, *Bax*, *Caspase9*, and *Cyt-c* in lung tissues were measured by RT-qPCR. B, C) Protein levels of Bcl2, Bax, Caspase9, and Cyt-c in lung tissues were analyzed by Western blot. D) The mRNA expression levels of *Tnf-α* and *Il-6* in lung tissues were measured by RT-qPCR. E, F) Intracellular ROS levels were measured using the DCFH-DA fluorescent probe (scale bar: 100 μm). Data were presented as mean ± SD (*n* = 5). One-way analysis of variance (ANOVA) followed by Tukey's post hoc test were used for statistical analysis.

RT-qPCR results demonstrated that R13A-MOTS-c down-regulated mRNA expression levels of pro-apoptotic genes (*Bax*, *Caspase9*, and *Cyt-c*) while up-regulating the anti-apoptotic gene *Bcl2*. However, this protective effect was abolished when cells were pre-treated with JPH203 (Fig. 11A). The changes in apoptosis-related proteins mirrored the alterations in mRNA levels (Fig. 11B and C). Additionally, the mRNA expression levels of *Tnf-α* and *Il-6* were not reduced by R13A-MOTS-c after JPH203 treatment (Fig. 11D). DCFH-DA assay results indicated that the inhibitory effect of R13A-MOTS-c on ROS in MLE-12 cells was also abolished by JPH203 (Fig. 11E and F). These findings collectively suggest that inhibition of LAT1 eliminated the protective role of R13A-MOTS-c against radiation-induced damage in MLE-12 cells.

## 3 Discussion

RILI is a common complication of thoracic tumor radiotherapy, severely impacting patients' quality of life and treatment outcomes, presenting an urgent challenge for both clinical practices. Our study suggests that replacing the 13th arginine in MOTS-c with alanine to generate R13A-MOTS-c significantly enhances its membrane permeability. In a RILI mouse model, R13A-MOTS-c effectively alleviated inflammation, oxidative stress, and mitochondrial damage in lung tissue. *In vitro* experiments further confirmed that R13A-MOTS-c penetrates the cell membrane by binding to the membrane protein LAT1, activating transcription factors Nrf2 and mitigating damage in lung epithelial cells. The LAT1 inhibitor JPH203 reversed its protective effects. The study indicates that R13A-MOTS-c alleviates RILI by targeting LAT1, highlighting its potential as a novel therapeutic agent for RILI.

In metabolic diseases, the levels of MOTS-c are significantly reduced. For instance, serum MOTS-c levels are significantly lower in patients with type 2 diabetes, which may be associated with insulin resistance and metabolic disorders [27]. Similarly, obese individuals show a notable decrease in MOTS-c levels, suggesting its potential role in energy metabolism and fat accumulation [28]. In atherosclerosis patients, reduced MOTS-c levels may be linked to vascular endothelial dysfunction and increased oxidative stress [29]. Heart failure patients also demonstrate decreased MOTS-c levels, indicating its possible involvement in myocardial cell energy metabolism and functional maintenance [30]. In neurodegenerative diseases, reduced MOTS-c levels in Alzheimer's patients may be associated with neuronal mitochondrial dysfunction and increased oxidative stress [31]. Furthermore, MOTS-c levels decline significantly with age, likely reflecting its role in maintaining mitochondrial function and antioxidant capacity [32]. In cancer patients, reduced MOTS-c levels may correlate with tumor cell metabolic reprogramming and mitochondrial dysfunction [33]. These findings align with our results, peripheral blood MOTS-c levels were significantly reduced in clinical samples of RILI. Collectively, these observations suggest that MOTS-c may play a regulatory role in physiological functions across multiple disease states.

Studies have suggested that MOTS-c exhibits significant cytoprotective effects in multiple disease models. Specifically, MOTS-c alleviates inflammation, oxidative stress, and endothelial cell damage in pulmonary vascular endothelial cells caused by ischemia-reperfusion [34]. Moreover, it reduces LPS-induced neutrophil infiltration and inhibits the release of inflammatory cytokines such as TNF-α, IL-1β, and IL-6 [35]. In acute lung injury models, N-formylmethionyl-leucyl-phenylalanine disrupts the vascular endothelial barrier through fpr2-mediated iron deposition, exacerbating ALI progression. However, MOTS-c demonstrates protective therapeutic potential by mitigating N-formylmethionyl-leucyl-phenylalanine-induced damage [36]. Further research indicates that MOTS-c protects against ALI induced by myocardial ischemia-reperfusion by inhibiting iron deposition, an effect dependent on the peroxisome proliferator-activated receptor gamma signaling pathway [37]. Additionally, our previous findings revealed that MOTS-c at 10 mg/kg alleviated RILI [4].

MOTS-c has been shown to exert its biological functions by activating transcription factors, but its effects depend on its ability to penetrate cell membranes. However, as a water-soluble peptide, MOTS-c has limited membrane permeability due to its hydrophilic properties, particularly the 13th and 16th arginine residue (hydrophilicity index of −0.938), which restricts its membrane penetration. Studies show that replacing this arginine with alanine (R13A-MOTS-c and R16A-MOTS-c) increases the peptide's average hydrophilicity coefficient from −0.938 to −0.544, significantly enhancing its membrane permeability. Notably, although R16A-MOTS-c shares a comparable increase in hydrophobicity index with R13A-MOTS-c, immunofluorescence results showed that the membrane permeability of R16A-MOTS-c was not enhanced. This observation suggests that changes in hydrophobicity are not the sole determinant of membrane penetration capability; rather, the position of the mutation may affect peptide conformation, charge distribution, transporter recognition efficiency, and even intracellular stability [38]. Overall, the R13A modification opens new avenues for the application of MOTS-c in disease treatment.

LAT1 is a transporter that facilitates the entry of various amino acids into cells in a Na^+^ and pH-independent manner. It demonstrates a strong ability to specifically recognize and transport exogenous peptides without endosomal/lysosomal engulfment or degradation [39]. Recent studies have found that LAT1 can transport proteins of different molecular weights and isoelectric points, such as cytochrome C (molecular weight 12.4 kDa), β-galactosidase (molecular weight 430 kDa), egg serum albumin (isoelectric point 4.5), lysozyme (isoelectric point 10.8), ribonuclease A, superoxide dismutase, trypsin, CRISPR-Cas9 nuclease, horseradish peroxidase, and saporin, into tumor cells and nerve cells, where they may subsequently exert biological effects [40,41]. In this study, immunofluorescence analysis and molecular docking experiments indicate that the co-localization of MOTS-c with LAT1 mediates its cellular uptake. The enhanced cellular uptake of R13A-MOTS-c may be attributed to its increased binding affinity to LAT1. Consistent with this, the differential uptake between R13A-MOTS-c and R16A-MOTS-c strongly suggests a position-specific recognition mechanism by LAT1, rather than one depends solely on overall hydrophobicity. Furthermore, the protective effects of R13A-MOTS-c against oxidative stress, inflammation, and mitochondrial function in MLE-12 cells were significantly reduced upon treatment with the LAT1 inhibitor JPH203, further supporting the role of LAT1 in mediating its functional activity.

Accumulating evidence indicates that MOTS-c exerts cytoprotective effects by activating the transcription factor Nrf2. MOTS-c has been shown to mitigate vascular endothelial cell apoptosis triggered by pulmonary ischemia-reperfusion through Nrf2 activation [34]. In a mouse model hypoxia-induced intrauterine growth restriction, MOTS-c protects the placenta from injury via Nrf2 signaling [42]. Moreover, in allergic asthma, MOTS-c alleviates airway barrier dysfunction by inhibiting Nrf2 pathway-related epithelial cell apoptosis [43]. Consistent with these observations, the present study demonstrates that R13A-MOTS-c exerts protective effects at least partially through activating Nrf2, which was further validated using primary lung epithelial cells from Nrf2-knockout mice. As a transcription factor, Nrf2 exerts its biological functions by activating downstream target genes such as HO-1, NQO1 and GCLC [44]. However, in this study, only HO-1 expression was assessed as a downstream indicator of Nrf2 activation, whereas the expression of NQO1 and GCLC was not examined. This represents a limitation of the current work regarding the comprehensive analysis of Nrf2-mediated signaling, which warrants further investigation in future studies.

This study provides valuable insights, yet several limitations should be acknowledged. LAT1-knockout mice were not used to directly validate LAT1's essential role in MOTS-c transport. Moreover, the protective effects of R13A-MOTS-c against radiation injury in human primary lung epithelial cells require further investigation. In addition, our murine model utilized high-dose single-fraction irradiation, which differs from the standard fractionated low-dose radiotherapy protocols used clinically. In future studies, we will establish a multiple low-dose irradiation mice model to further investigate the radioprotective effects of R13A-MOTS-c.

**Fig. 12:**
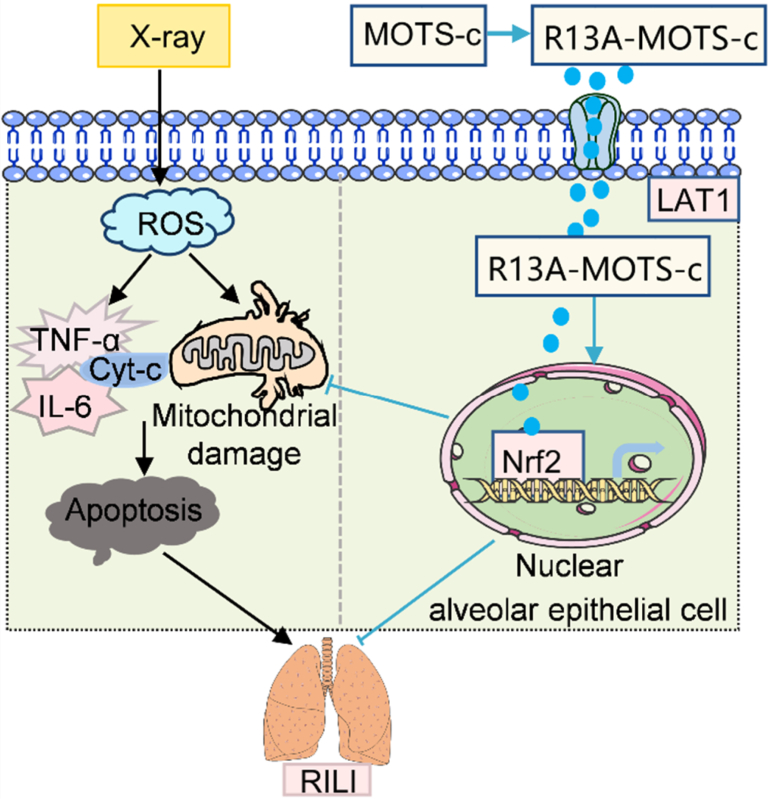
Graphical abstract. LAT1-mediated uptake of R13A-MOTS-c alleviates radiation-induced lung injury through Nrf2 pathway activation and mitochondrial function restoration, offering a promising therapeutic strategy for clinical applications.

In conclusion, this study indicates that the R13A-MOTS-c peptide effectively reduces mitochondrial ROS generation, maintains mitochondrial homeostasis, inhibits epithelial cell mitochondrial apoptosis, and significantly enhances the bioavailability of MOTS-c by targeting the LAT1 membrane protein, highlighting its potential as a novel radioprotective agent (Fig. 12).

## 4 Experimental section

*Collection of Serum from RILI Patients:* Collect serum samples from patients who underwent radiotherapy at Jiangnan University Hospital between January 2021 and December 2022. We collected serum samples from 10 thoracic cancer patients who developed RILI after radiotherapy (RILI) and 10 thoracic cancer patients who did not develop RILI after radiotherapy as controls. The basic characteristics of the patients were shown in Table S1.

*Bioinformatics Analysis of MOTS-c Polypeptide:* Multiple sequence alignment was performed using WebLogo 3 across 14 species, including Human, Chimpanzee, Bonobo, Orangutan, Mouse, Rat, Naked Mole Rat, Dog, Cow, Zebrafish, Lion, Bear, Horse, and Dolphin. The Grand Average of Hydropathicity (GRAVY) was predicted for three polypeptide molecules using ProtParam (https://web.expasy.org/protparam). The GRAVY score for a full-length peptide was calculated using the following formula: $GRAVY=\frac{1}{N}\sum_{i=1}^{N}H_{i}\text{.}$ For fluorescence labeling, the 14th lysine (Lys) residue was conjugated with FITC. Additionally, arginine residues at positions 13 and 16 were substituted with alanine (Ala) to minimize potential interference with molecular interactions.

*Acquisition of the peptides:* MOTS-c, R13A-MOTS-c, R16A-MOTS-c, FITC-MOTS-c, FITC-R13A-MOTS-c, and FITC-R16A-MOTS-c were synthesized by Wuxi Maimoto Biological Technology Co., Ltd., with purities exceeding 95%. Due to the high conservation of the first 11 amino acids in MOTS-c, we selected the C-terminal five residues for modification. The 14th amino acid is an arginine, whose side chain contains a primary amine group. This amine group exhibits high chemical reactivity, making it a classical site for FITC labeling.

*Molecular Modeling and Docking:* The LTA1, MOTS-c and R13A-MOTS-c protein sequences were retrieved from the UniProt database. The sequences were submitted to the AlphaFold server for protein structure prediction and interaction analysis. The predicted interaction models were visualized using PyMOL (PyMOL Molecular Graphics System, version 3.0.4, Schrodinger, New York, United States), in which the polar interactions between proteins were highlighted to illustrate the key binding sites [45].

*Animal Experiment:* Male C57BL/6 mice aged 6-8 weeks were randomly assigned to four experimental groups (*n* = 5): Normal group (Normal), Irradiation group (IR), MOTS-c intervention group (IR + MOTS-c (5 mg/kg)), and R13A-MOTS-c intervention group (IR + R13A-MOTS-c (5 mg/kg)). The irradiation experiment was conducted at the Radiation Center of Jiangnan University Affiliated Hospital in Wuxi (Varian Vital Beam Linear Accelerator). After anesthesia, mice were exposed to whole-lung irradiation with a total dose of 20 Gy at a rate of 5 Gy/min [46]. For treatment, mice received daily intraperitoneal injections of MOTS-c or R13-MOTS-c (5 mg/kg), beginning 2 h before irradiation and continuing for 13 days thereafter. Two weeks post-irradiation, the mice were euthanized, and blood, bronchoalveolar lavage fluid, and lung tissue samples were collected for subsequent experiments. All animal experiments were approved by the Ethics Committee of Jiangnan University (JN.No20240330c0351031 [162]).

*Histological Analysis:* Lung tissues were fixed in 4% paraformaldehyde, followed by dehydration, clearing, and paraffin embedding. The embedded tissues were sectioned into 4 μm thick slices. After dewaxing, the sections were hydrated through a gradient alcohol series and then subjected to hematoxylin and eosin (HE) staining. The staining results were scanned using the Pannoramic MIDI (3D HISTECH, Budapest, Hungary).

*Bronchoalveolar Lavage Fluid:* After anesthetizing the mice, the trachea was surgically exposed and cannulated. Pre-cooled 0.6 mL PBS was slowly instilled into the lungs via tracheal cannula, followed by gentle aspiration of the lavage fluid. This procedure was repeated three consecutive times. Subsequently, the collected lavage fluid was centrifuged (4 °C, 500×*g*, 5 min), and the supernatant was collected for protein measurement while the pellet was resuspended for cell counting.

*Cell Culture and Treatment:* Mouse lung epithelial cells MLE-12 (ATCC, USA) were exposed to 8 Gy of X-ray irradiation [47]. The cells were pretreated with MOTS-c and R13A-MOTS-c 2 h prior to irradiation. After 24 h post-irradiation, cells were harvested for subsequent experiments. The final concentrations of the drugs used for cell treatment were as follows: MOTS-c (5 μM), R13A-MOTS-c(5 μM), and JPH203 (25 μM) respectively [48].

*Biochemical Indexes Analysis:* The serum levels of LDH, MPO, MDA, SOD, and GSH were quantified using commercial assay kits according to the manufacturers' protocols, while cellular contents of LDH, GSH, SOD, and MDA were similarly analyzed with their corresponding kits.

*CCK8 Assay:* MLE-12 cells were seeded in 96-well plates at a density of 8 × 10^4^ cells/mL. After the cells adhered, they were pretreated with varying concentrations of MOTS-c and R13A-MOTS-c (10, 9, 8, 7, 6, 5, 4, 3, 2, and 1 μM) for 2 h. Subsequently, the cells were exposed to 8 Gy of X-ray irradiation. After 24 h post-irradiation, 10 μL of CCK8 reagent was added to each well, and the plates were incubated for an additional 2 h. The absorbance of each well was then measured at a wavelength of 450 nm using a microplate reader.

*Real-Time Quantitative Polymerase Chain Reaction (RT-qPCR):* Total RNA was extracted from lung tissues or cells using Trziol, and the concentration and purity of RNA in the samples were measured using Nonadrop. The isolated RNA was then reverse-transcribed into cDNA following the instructions of the PrimeScript RT reagent kit. Subsequently, real-time PCR was performed using SYBR Premix Ex TaqTM and the LightCycler® 480 system to detect the expression of target mRNA. The relative expression levels of the target genes were calculated using the 2^−ΔΔCt^ quantification method. The primer sequences for RT-qPCR are listed in Table S2.

*ELISA Assay:* The concentration of MOTS-c in human serum was measured using a commercial ELISA kit (CEX132Hu; Cloud-Clone Corp., USA). Briefly, 50 μL of standards, blank, or samples were added to designated wells, followed immediately by 50 μL of Detection Reagent A. The plate was sealed and incubated at 37 °C for 1 h. After aspiration, wells were washed three times with 350 μL of Wash Solution (1–2 min soak each). Subsequently, 100 μL of Detection Reagent B was added, and the plate was incubated at 37 °C for 30 min. After five additional washes, 90 μL of Substrate Solution was added and incubated in the dark at 37 °C for 10–20 min. The reaction was stopped by adding 50 μL of Stop Solution, and the absorbance was read immediately at 450 nm.

*Western Blot Assay:* Total protein was extracted from MLE-12 cells or lung tissues, and the protein concentration was determined. Equal amounts of protein samples were separated by 12% SDS-PAGE electrophoresis, and the proteins were then transferred onto a PVDF membrane. The membrane was blocked with 5% skim milk for 2 h, followed by incubation with primary antibodies at 4 °C overnight. After washing, the membrane was incubated with HRP-conjugated secondary antibodies at room temperature for 1 h. The membrane was washed again, and ECL chemiluminescent substrate was added. Protein bands were detected using an imaging system. Finally, the band intensity was analyzed using software such as ImageJ to calculate the relative expression levels of the target proteins. The primary antibodies are listed in Table S3.

*Small Interfering RNA (siRNA) Transfection*: MLE-12 cells were transfected with siRNA against mouse LAT1 (GenePharma, Shanghai, China) using GP-transfect-Mate transfection reagent (GenePharma, Shanghai, China), following a previously described protocol. The primer sequences of MOTS-c siRNA used in this study are presented in Table S4.

*LAT1 overexpression in MLE-12 cell:* MLE-12 cells were transfected with LAT1 overexpression plasmid (GenePharma, Shanghai, China) using GP-transfect-Mate transfection reagent (GenePharma, Shanghai, China), following a previously described protocol.

*Flow Cytometry:* MLE-12 cells were seeded in 6-well plates. After cell adhesion, the cells were pre-incubated with either BCH (2.5 mM) or leucine (1.5 mM) for 15 min, followed by co-incubation with FITC-labeled peptides (FITC-MOTS-c or FITC-R13A-MOTS-c) for 30 min [31]. The intracellular fluorescence intensity was then measured by flow cytometry to evaluate peptide uptake.

*Co-immunoprecipitation (Co-IP)*: MLE-12 cells were lysed to prepare protein extracts. The lysates were incubated overnight at 4 °C with a MOTS-c specific antibody, followed by the addition of Protein A/G beads to capture the antibody-target protein complexes along with any interacting partners. After washing to remove non-specifically bound components, the immunoprecipitated complexes were eluted and analyzed by Western blotting.

*Nuclear and Cytoplasmic Protein Extraction:*Nuclear and cytoplasmic proteins were extracted from MLE-12 cells using the Kangwei Century Bio-tech extraction kit according to the manufacturer's instructions. All procedures were performed with the addition of protease inhibitors and maintained at 4 °C. The extracted proteins were subsequently analyzed by Western blotting.

*Isolation of Primary alveolar Cells:* Pathogen-free male mice (8 weeks old) with a C57BL/6 background, including wild-type (WT) and Nrf2-knockout (Nrf2^−/−^) mice, were obtained from the Model Animal Research Center, MARC, Nanjing (project No. XM002783). Primary alveolar epithelial cells were isolated following the method described in previous literature [34].

*TdT-Mediated dUTP Nick-End Iabeling (TUNEL) Staining:* Cells and lung tissue sections were fixed with 4% paraformaldehyde, permeabilized, and incubated with TUNEL reaction mixture at 37 °C in the dark for 1 h. After washing away unbound reagents, the nuclei were stained with DAPI (Beyotime, Shanghai, China). The slides were then mounted and observed under a fluorescence microscope.

*Immunofluorescent Staining:* Cells were fixed with 4% paraformaldehyde at room temperature for 15 min, followed by permeabilization with 0.1% Triton X-100 for 20 min. After fixation, the cells were washed with PBS and blocked with 5% goat serum at room temperature for 30 min. The corresponding fluorescent secondary antibodies were incubated with the cells at room temperature. After washing with PBS, the cells were counterstained with DAPI reagent (Beyotime, Shanghai, China) and imaged using a Zeiss LSM 880 confocal fluorescence microscope.

*Detection of Cytosolic ROS and Mitochondrial ROS:* DCFH-DA (for detecting cytoplasmic ROS) or MitoSOX (for detecting mitochondrial ROS) staining probes were added to MLE-12 cells, followed by incubation at 37 °C in the dark for 20-30 min. Subsequently, the cells were washed 2-3 times with pre-warmed PBS to remove unbound probes. Subsequently, images were acquired using a fluorescence microscope.

*Mitochondrial Membrane Potential:* JC-1 staining probe was added to MLE-12 cells, followed by incubation at 37 °C for 30 min. Subsequently, the cells were washed 2-3 times with pre-warmed PBS to remove unbound probes. Fluorescence images were acquired using a fluorescence microscope.

*Intracellular Calcium Concentration in MLE-12 cells:* Rhod-2 AM Ca^2+^ fluorescent probe was added to MLE-12 cells, followed by incubation at 37 °C for 30 min. Subsequently, the cells were washed 2-3 times with pre-warmed PBS to remove unbound probes. Fluorescence images were acquired using a fluorescence microscope.

*Adenosine Triphosphate Content:* MLE-12 cells were lysed using lysis buffer to release ATP. Subsequently, the lysate was mixed with the ATP detection reagent and incubated at room temperature in the dark for 10 min. The chemiluminescence intensity was then measured using a microplate reader.

*Statistical Analysis:* Data analysis was performed using GraphPad Prism software, and all data are presented as mean ± standard deviation. Comparisons between two groups were conducted using the *t*-test, while comparisons among multiple groups were performed using one-way analysis of variance (ANOVA) followed by Tukey's post hoc test. *P*-value <0.05 was considered statistically significant.

## CRediT authorship contribution statement

**Yan-li Zhang:** Conceptualization, Data curation, Investigation, Methodology, Software, Writing – original draft, Writing – review & editing. **Guo Huang:** Formal analysis, Investigation, Methodology, Resources. **Sheng-peng Li:** Methodology, Software, Supervision. **Wen-long Zhang:** Methodology, Software, Visualization. **Dan Chen:** Data curation, Supervision. **Liu-gen Jin:** Conceptualization, Writing – original draft. **Qing-feng Pang:** Conceptualization, Funding acquisition, Supervision, Writing – original draft, Writing – review & editing. **Ya-xian Wu:** Data curation, Funding acquisition, Investigation, Software, Supervision, Writing – original draft, Writing – review & editing. **Jian-feng Huang:** Conceptualization, Formal analysis, Funding acquisition, Resources, Supervision, Writing – review & editing.

## Declaration of competing interest

The authors declare that they have no competing interests.

## Data Availability

No data was used for the research described in the article.

## References

[1] Barnett G.C., West C.M.L., Dunning A.M. (2009). Normal tissue reactions to radiotherapy: towards tailoring treatment dose by genotype. Nat. Rev. Cancer.

[2] Chang S., Lv J., Wang X. (2024). Pathogenic mechanisms and latest therapeutic approaches for radiation-induced lung injury: a narrative review. Crit. Rev. Oncol. Hematol..

[3] Rao X., Zhou D., Deng H. (2023). Activation of NLRP3 inflammasome in lung epithelial cells triggers radiation-induced lung injury. Respir. Res..

[4] Zhang Y., Huang J., Zhang Y. (2024). The mitochondrial-derived peptide MOTS-c alleviates radiation pneumonitis via an Nrf2-Dependent mechanism. Antioxidants (Basel).

[5] Ahluwalia A., Jones M.K., Hoa N. (2019). Mitochondria in gastric epithelial cells are the key targets for NSAIDs-induced injury and NGF cytoprotection. J. Cell. Biochem..

[6] Lee C., Zeng J., Drew B.G. (2015). The mitochondrial-derived peptide MOTS-c promotes metabolic homeostasis and reduces obesity and insulin resistance. Cell Metab..

[7] Kim K.H., Son J.M., Benayoun B.A. (2018). The mitochondrial-encoded peptide MOTS-c translocates to the nucleus to regulate nuclear gene expression in response to metabolic stress. Cell Metab..

[8] Benayoun B.A., Lee C. (2019). MOTS-c: a mitochondrial-encoded regulator of the nucleus. Bioessays.

[9] Zhong P., Peng J., Hu Y. (2022). Mitochondrial derived peptide MOTS-c prevents the development of heart failure under pressure overload conditions in mice. J. Cell Mol. Med..

[10] Yu W.D., Kim Y.J., Cho M.J. (2021). The mitochondrial-derived peptide MOTS-c promotes homeostasis in aged human placenta-derived mesenchymal stem cells in vitro. Mitochondrion.

[11] Wang M., Wang G., Pang X. (2022). MOTS-c repairs myocardial damage by inhibiting the CCN1/ERK1/2/EGR1 pathway in diabetic rats. Front. Nutr..

[12] Mohtashami Z., Singh M.K., Salimiaghdam N. (2022). MOTS-c, the Most recent mitochondrial derived peptide in human aging and age-related diseases. Int. J. Mol. Sci..

[13] Kim S.-J., Miller B., Kumagai H. (2021). Mitochondrial-derived peptides in aging and age-related diseases. GeroScience.

[14] Shi M., Jiang Z., Xiao Y. (2022). Stapling of short cell-penetrating peptides for enhanced tumor cell-and-tissue dual-penetration. Chem Commun (Camb).

[15] Jiang J., Chang X., Nie Y. (2023). Orally administered MOTS-c analogue ameliorates dextran sulfate sodium-induced colitis by inhibiting inflammation and apoptosis. Eur. J. Pharmacol..

[16] Tai W., Zhao P., Gao X. (2020). Cytosolic delivery of proteins by cholesterol tagging. Sci. Adv..

[17] Huttunen J., Kronenberger T., Montaser A.B. (2023). Sodium-dependent neutral amino acid transporter 2 can serve as a tertiary carrier for l-Type amino acid transporter 1-Utilizing prodrugs. Mol. Pharm..

[18] Hou X., Song S., Xu Z. (2024). Prolactin upregulates amino acid uptake in dairy cow mammary epithelial cells via LAT1. J. Dairy Sci..

[19] LE Vee M., Jouan E., Lecureur V. (2016). Aryl hydrocarbon receptor-dependent up-regulation of the heterodimeric amino acid transporter LAT1 (SLC7A5)/CD98hc (SLC3A2) by diesel exhaust particle extract in human bronchial epithelial cells. Toxicol. Appl. Pharmacol..

[20] Guo H., Xu W., Nomoto T. (2023). Polymeric ligands comprising sulfur-containing amino acids for targeting tumor-associated amino acid transporters. Biomaterials.

[21] Tampio J., Markowicz-Piasecka M., Montaser A. (2022). L-type amino acid transporter 1 utilizing ferulic acid derivatives show increased drug delivery in the mouse pancreas along with decreased lipid peroxidation and prostaglandin production. Mol. Pharm..

[22] Venteicher B., Merklin K., Ngo H.X. (2021). The effects of prodrug size and a carbonyl linker on l-Type amino acid transporter 1-Targeted cellular and brain uptake. ChemMedChem.

[23] O'Kane R.L., ViñA J.R., Simpson I. (2004). Na+ -dependent neutral amino acid transporters A, ASC, and N of the blood-brain barrier: mechanisms for neutral amino acid removal. Am. J. Physiol. Endocrinol. Metab..

[24] Verrey F., Jack D.L., Paulsen I.T. (1999). New glycoprotein-associated amino acid transporters. J. Membr. Biol..

[25] Verrey F., Meier C., Rossier G. (2000). Glycoprotein-associated amino acid exchangers: broadening the range of transport specificity. Pflügers Archiv.

[26] Yan R., Zhao X., Lei J. (2019). Structure of the human LAT1-4F2hc heteromeric amino acid transporter complex. Nature.

[27] Ramanjaneya M., Bettahi I., Jerobin J. (2019). Mitochondrial-derived peptides are Down regulated in diabetes subjects. Front Endocrinol (Lausanne).

[28] Luo Y.-H., Xie L., Li J.-Y. (2023). Serum MOTS-C levels are decreased in obese children and associated with vascular endothelial function. Diabetes Metab. Syndr. Obes..

[29] Li Y., Li Z., Ren Y. (2024). Mitochondrial-derived peptides in cardiovascular disease: novel insights and therapeutic opportunities. J. Adv. Res..

[30] Bigazzi F., Adorni M.P., Puntoni M. (2017). Analysis of serum cholesterol efflux capacity in a minipig model of nonischemic heart failure. J. Atherosclerosis Thromb..

[31] Miller B., Kim S.-J., Kumagai H. (2022). Mitochondria-derived peptides in aging and healthspan. J. Clin. Investig..

[32] Son J.M., Lee C. (2019). Mitochondria: multifaceted regulators of aging. BMB Rep..

[33] Yin Y., Li Y., Ma B. (2024). Mitochondrial-derived peptide MOTS-c suppresses ovarian cancer progression by attenuating USP7-Mediated LARS1 deubiquitination. Adv. Sci. (Weinh.).

[34] Wang D.-D., Xu B., Sun J.-J. (2025). MOTS-c mimics remote ischemic preconditioning in protecting against lung ischemia-reperfusion injury by alleviating endothelial barrier dysfunction. Free Radic. Biol. Med..

[35] Xinqiang Y., Quan C., Yuanyuan J. (2020). Protective effect of MOTS-c on acute lung injury induced by lipopolysaccharide in mice. Int. Immunopharmacol..

[36] Wen Z., Fan J., Zhan F. (2024). The role of FPR2-mediated ferroptosis in formyl peptide-induced acute lung injury against endothelial barrier damage and protective effect of the mitochondria-derived peptide MOTS-c. Int. Immunopharmacol..

[37] Lu P., Li X., Li B. (2023). The mitochondrial-derived peptide MOTS-c suppresses ferroptosis and alleviates acute lung injury induced by myocardial ischemia reperfusion via PPARγ signaling pathway. Eur. J. Pharmacol..

[38] Pašalić L., Pem B., Jakas A. (2025). Peptide interaction with mixed lipid bilayers alters packing and hydrocarbon chain conformations. J. Liposome Res..

[39] Huttunen J., Agami M., Tampio J. (2021). Comparison of experimental strategies to study l-Type amino acid transporter 1 (LAT1) utilization by ligands. Molecules.

[40] Zhao Z., Liu X., Hou M. (2022). Endocytosis-independent and cancer-selective cytosolic protein delivery via reversible tagging with LAT1 substrate. Adv. Mater..

[41] Bahrami K., JäRVINEN J., Laitinen T. (2023). Structural features affecting the interactions and transportability of LAT1-Targeted phenylalanine drug conjugates. Mol. Pharm..

[42] Chen D., Zhao H.-M., Sun X.-L. (2025). MOTS-c protects against placental injury via Nrf2 activation in hypoxia-induced intrauterine growth restriction mice. Int. J. Mol. Med..

[43] Zhang W., Li S., Zhang Y. (2025). MOTS-c attenuates airway barrier dysfunction in allergic asthma by inhibiting epithelial apoptosis via Nrf2 pathway. Int. Immunopharmacol..

[44] Jakubowska M., Costa V.M., Krzeptowski W. (2025). Altered NRF2 signalling in systemic redox imbalance: insights from non-communicable diseases. Redox Biol..

[45] Guo C., Liu X., Mei Z. (2025). AMBP protects against aortic valve calcification by inhibiting ERK1/2 and JNK pathways mediated by FHL3. Theranostics.

[46] Zhang Y., Huang J., Li S. (2024). Pyrroloquinoline quinone alleviates mitochondria damage in radiation-induced lung injury in a MOTS-c-Dependent manner. J. Agric. Food Chem..

[47] Fleury H., Maceachern M.K., Stiefel C.M. (2023). The APE2 nuclease is essential for DNA double-strand break repair by microhomology-mediated end joining. Mol. Cell.

[48] Rivera C.N., Smith C.E., Draper L.V. (2023). The selective LAT1 inhibitor JPH203 enhances mitochondrial metabolism and content in insulin-sensitive and insulin-resistant C2C12 myotubes. Metabolites.

