# Supplementary material for: LAT1-mediated delivery of engineered R13A-MOTS-c attenuates radiation-induced lung injury via Nrf2 activation and mitochondrial protection
Source: Redox Biol. 2026 May 9;94:104204. doi: 10.1016/j.redox.2026.104204 (PMC13199819; doi:10.1016/j.redox.2026.104204)
Supplement: Multimedia component 1 [file mmc1.docx]

**Supplementary Figures and Tables**

**
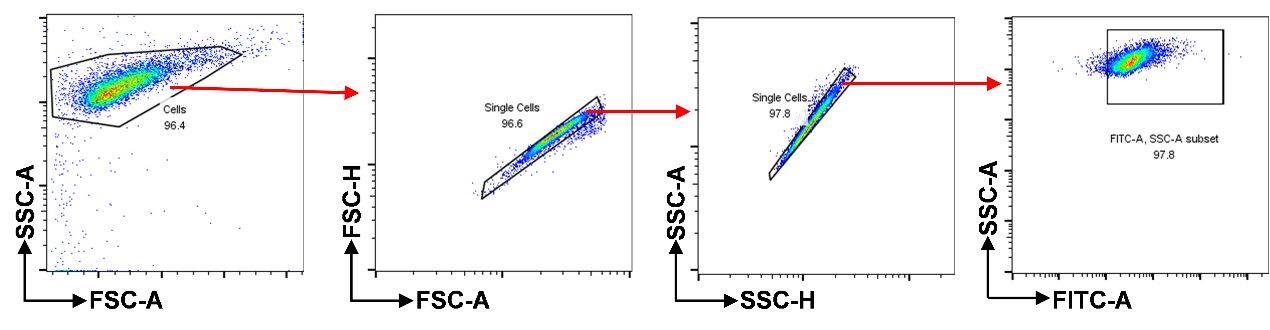
**

Figure S1. Flow cytometry gating strategy. Viable cells were first gated in the FSC/SSC dot plot, followed by sequential doublet exclusion using FSC-A/FSC-H and SSC-A/SSC-H dot plots to obtain single cells. Finally, the positive population was gated in the FITC histogram.

**
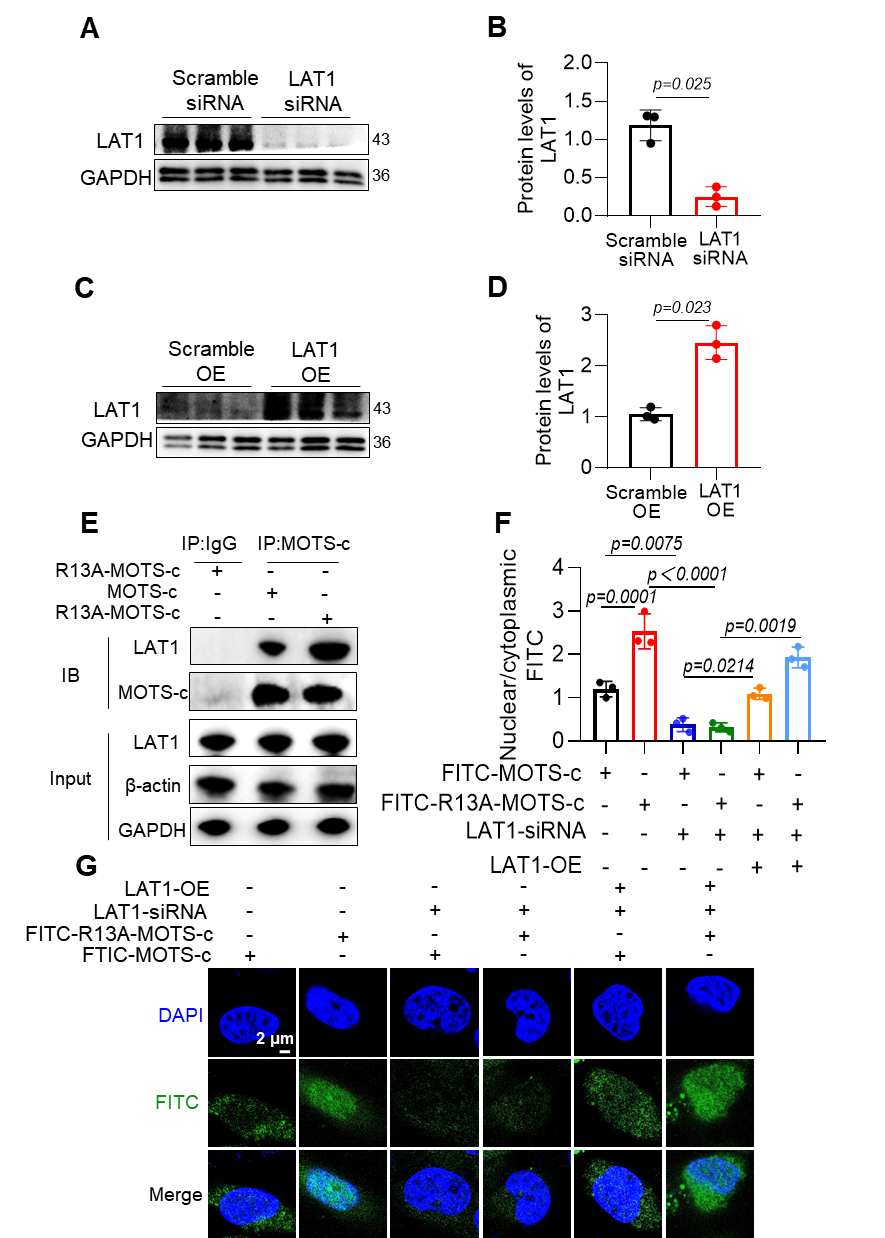
**

Figure S2. Genetic knockout and overexpression of LAT1 in MLE-12 cells. A–D) LAT1 protein expression levels in MLE-12 cells were assessed by Western blot analysis. E) The binding ability of MOTS-c and MOTS-cR13A to LAT1 was observed by Co-IP assay. F, G) Nuclear localization of MOTS-c assessed by confocal microscopy (scale bar: 2 μm). Data were presented as mean ± SD (n=3). One-way analysis of variance (ANOVA) followed by Tukey's post hoc test were used for statistical analysis.


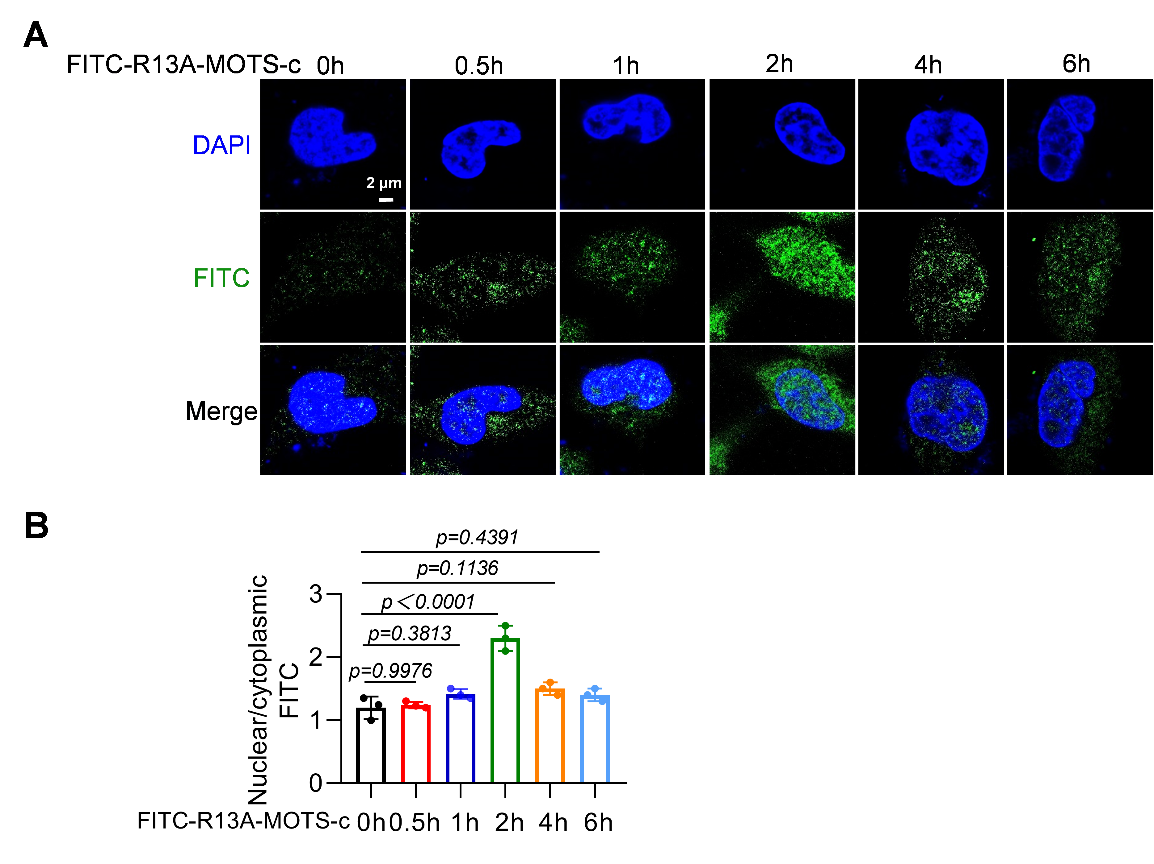


Figure S3. Optimization of pretreatment duration for R13A-MOTS-c in MLE-12 cells. A, B) Nuclear localization of MOTS-c assessed by confocal microscopy (scale bar: 2 μm). Data are presented as mean ± SD (*n*=3). ANOVA followed by Tukey's post hoc test were used for statistical analysis.


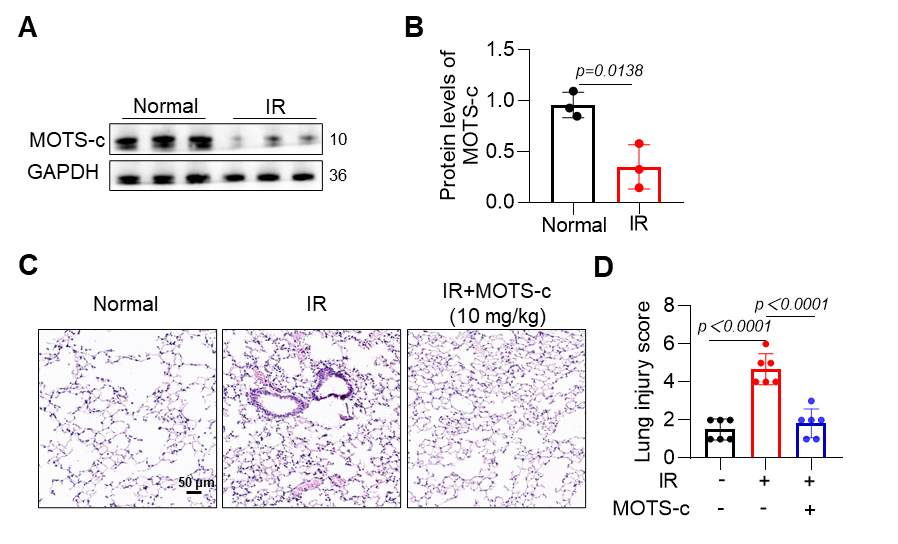


Figure S4. MOTS-c relieved radiation-induced inflammatory response in RILI mice. A-B) Protein levels of MOTS-c in lung tissues of RP mice were measured by Western blot. Data are presented as mean ± SD (*n*=3). Student’s *t*-test were used for statistical analysis. C, D) Lung tissue sections were stained with HE, scale bar = 50 μm. Data were presented as mean ± SD (*n*=6). One-way analysis of variance (ANOVA) followed by Tukey's post hoc test were used for statistical analysis.


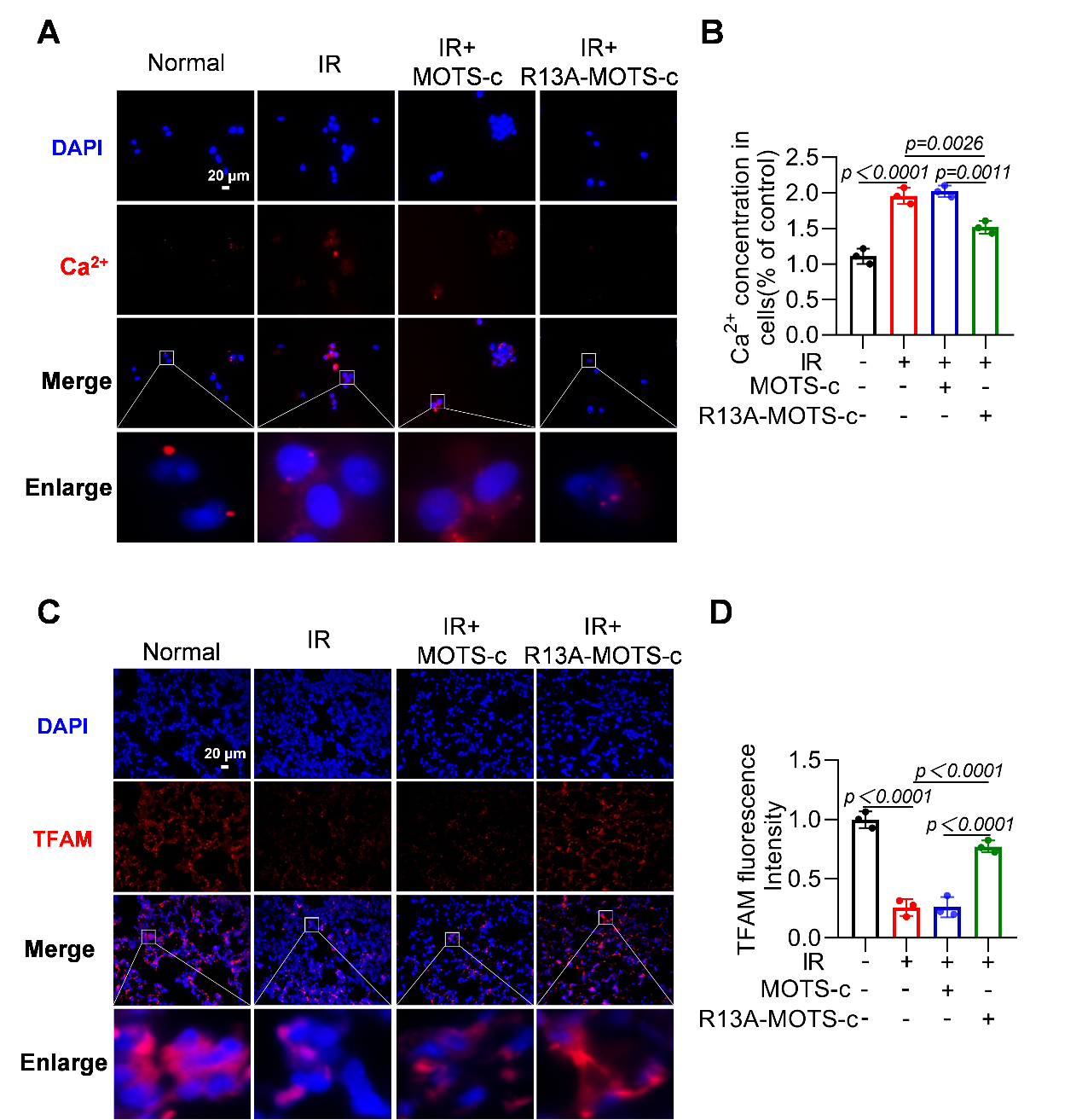


Figure S5. R13A-MOTS-c relieved radiation-induced inflammatory response in MLE-12 cell and RILI mice. A, B) Rhod-2, AM Red fluorescent probe was used to detect calcium content in MLE-12 (scale bar: 20 µm) (*n*=3). C, D) Lung tissue sections were stained with hematoxylin-eosin (HE), scale bar = 50 μm. Data were presented as mean ± SD (*n*=3). One-way analysis of variance (ANOVA) followed by Tukey's post hoc test were used for statistical analysis.

**
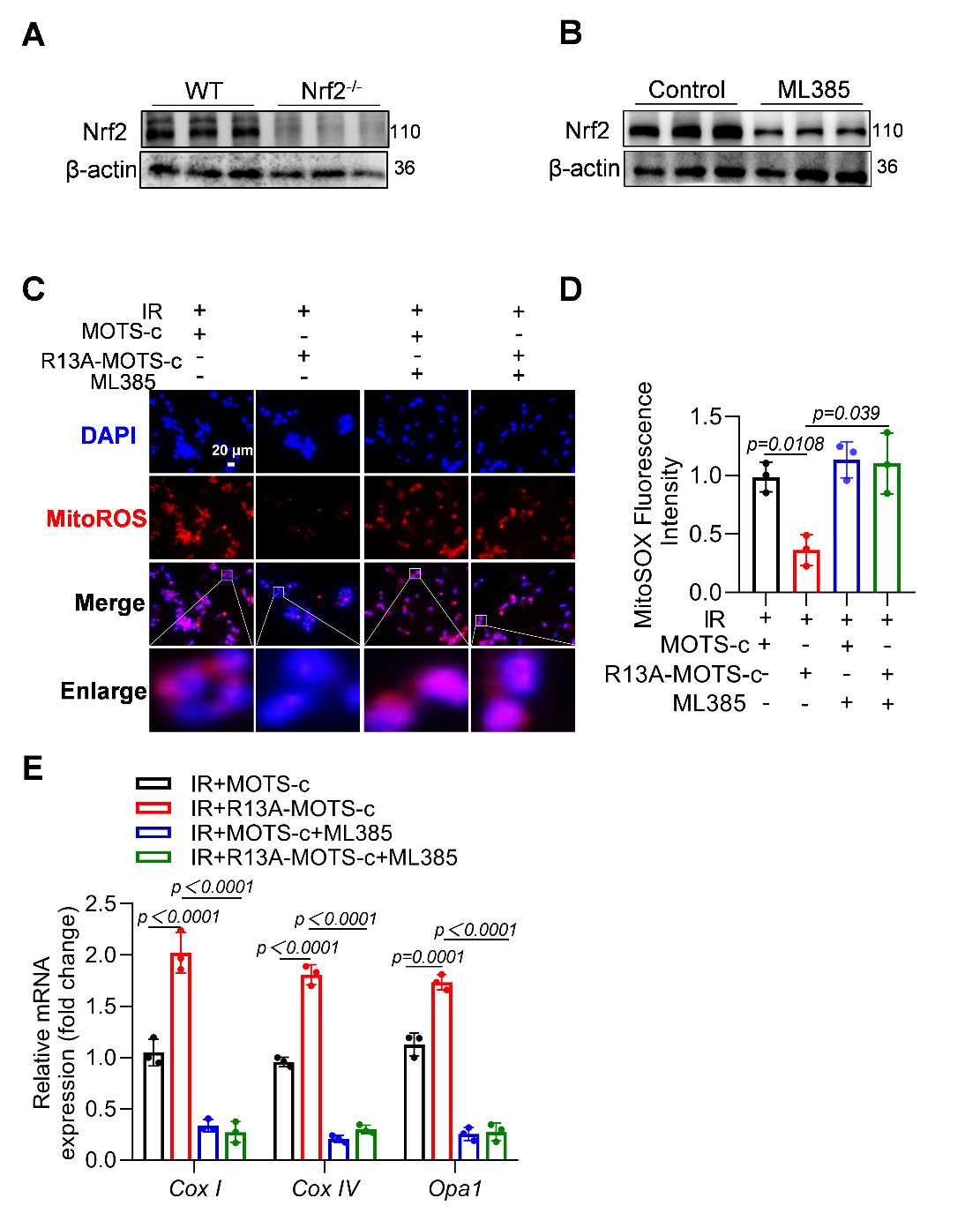
**

Figure S6. Engineered R13A-MOTS-c exerted radioprotection through an Nrf2-dependent mechanism. A) Protein levels of Nrf2 in WT and Nrf2^-/-^ mice were analyzed by Western blot. B) Protein levels of Nrf2 in ML385-treated MLE-12 cells were analyzed by Western blot. C, D) The mtROS levels in MLE-12 cells were detected using the MitoSOX Red fluorescent probe (scale bar: 20 µm). E) The mRNA levels of *Cox I, Cox IV and Opa1* in MLE-12 cells were determined by RT-qPCR. Data were presented as mean ± SD (*n*=3). One-way analysis of variance (ANOVA) followed by Tukey's post hoc test were used for statistical analysis.


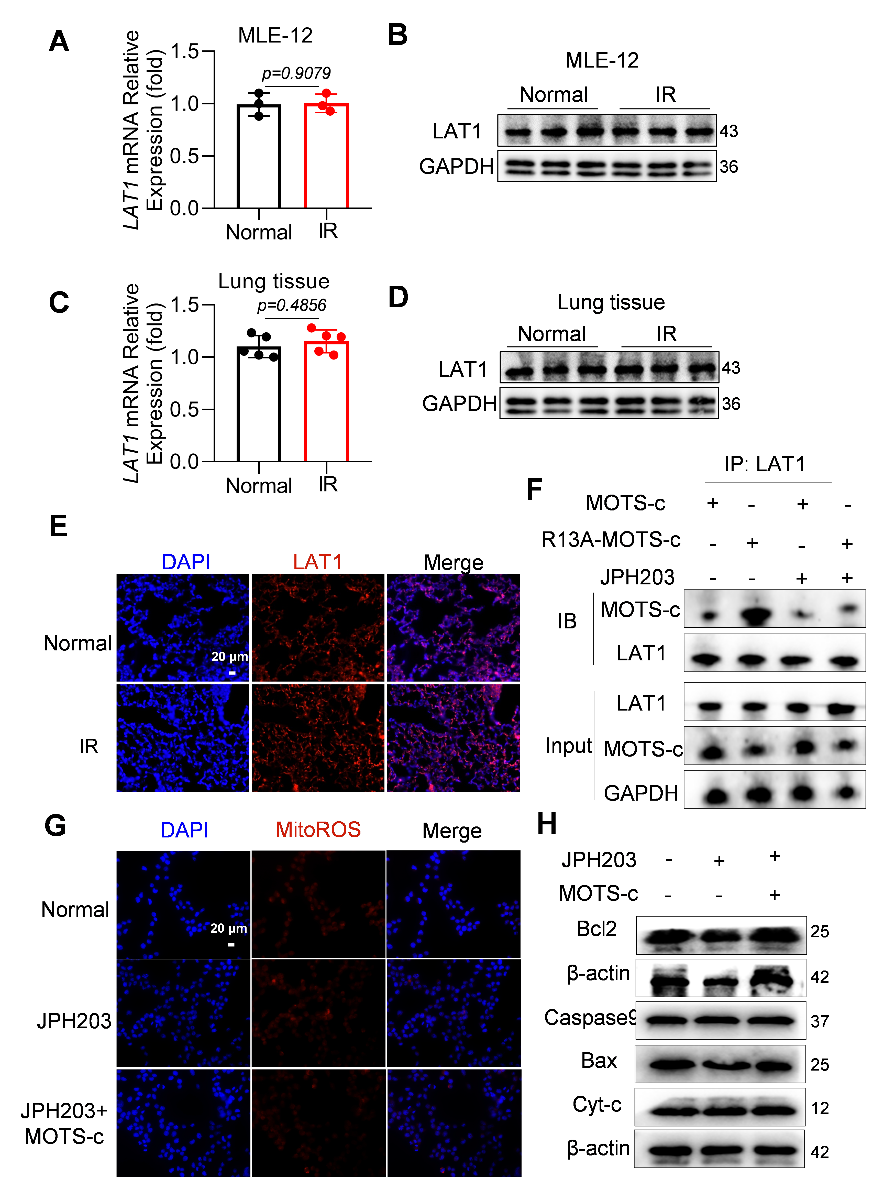


Figure S7. Radiation exposure did not alter LAT1 protein levels, and neither MOTS-c nor R13A-MOTS-c affected the binding of JPH203 to LAT1. A) mRNA expression levels of *LAT1* in MLE-12 cells were quantified by RT-qPCR (*n*=3). B) Protein levels of LAT1 in MLE-12 cells were analyzed by Western blot (*n*=3). C) The mRNA expression levels of *LAT1* in lung tissue were measured by RT-qPCR (*n*=5). D) Protein levels of LAT1 in lung tissue were analyzed by Western blot (*n*=3). E) Immunofluorescence analysis of lung tissues stained with LAT1 (red) and DAPI (blue) (scale bar: 20 μm, *n*=3). F) The binding ability of MOTS-c, MOTS-cR13A and JPH203 to LAT1 was detected by Co-IP assay. G) The mtROS levels in primary lung epithelial cells were detected using the MitoSOX Red fluorescent probe (scale bar: 20 µm). H) Protein levels of Bcl2, Bax, Caspase9 and Cyt-c in lung tissues were analyzed by Western blot. Data are presented as mean ± SD. Comparisons between two groups were conducted using the t-test, while comparisons among multiple groups were performed using one-way analysis of variance (ANOVA) followed by Tukey's post hoc test.

Table S1 Clinical data of the study patients

| **Characteristic** |  | **Control（*n*=10）** | **RILI（*n*=10）** |
| --- | --- | --- | --- |
| Age (years) | Median  Range | 67  50-75 | 68  65-74 |
| Sex | Male  Female | 9  1 | 10  0 |
| Smoking | Yes  No | 6  4 | 5  5 |
| RILI stage | 2  3 | 0  0 | 5  5 |

Table S2 The primer sequences for RT-qPCR

| Gene | Primer | Sequence (5’-3’) |
| --- | --- | --- |
| *Gapdh* | Forward Primer | TGGCCTTCCGTGTTCCTAC |
|  | Reverse Primer | GAGTTGCTGTTGAAGTCGCA |
| *Nrf2* | Forward Primer | TCTTGGAGTAAGTCGAGAAGTGT |
|  | Reverse Primer | GTTGAAACTGAGCGAAAAAGGC |
| *Bax* | Forward Primer  Reverse Primer | AGACAGGGGCCTTTTTGCTAC  AATTCGCCGGAGACACTCG |
| *Bcl2* | Forward Primer  Reverse Primer | GAGAGCGTCAACAGGGAGATG  CCAGCCTCCGTTATCCTGGA |
| *Caspase9* | Forward Primer  Reverse Primer | GGCTGTTAAACCCCTAGACCA  TGACGGGTCCAGCTTCACTA |
| *Il-6* | Forward Primer  Reverse Primer | ACTTCCATCCAGTTGCCTTCTTGG  TTAAGCCTCCGATTGTGAAGTG |
| *Tnf-α* | Forward Primer  Reverse Primer | CCTGTAGCCCACGTCGTAG  GGGAGTAGACAAGGTACAACCC |
| *Cox I* | Forward Primer  Reverse Primer | GAAGAGACAGTGTTTCATGTGGTGT  TCCTGGGCCTTTCAGGAATA |
| *Cox IV* | Forward Primer  Reverse Primer | ATTGGCAAGAGAGCCATTTCTAC  TGGGGAAAGCATAGTCTTCACT |
| *Opa1* | Forward Primer  Reverse Primer | CGACTTTGCCGAGGATAGCTT  CGTTGTGAACACACTGCTCTTG |

Table S3 List of primary antibodies used in Western blots

| **Antibody** | **Dilution** |
| --- | --- |
| Nrf2 (#12721, rabbit monoclonal, Cell Signaling Technology)  OPA1 (#66583-1-Ig, mouse monoclonal, Proteintech)  COX I (#0807-3, rabbit polyclonal, HUABIO)  COX IV (#ET1701-63, rabbit monoclonal, HUABIO)  Bcl2 (#ER1802-97, rabbit polyclonal, HUABIO)  Bax (#ET1603-34, rabbit monoclonal, HUABIO)  Cyt-c (#ET1610-16, rabbit monoclonal, HUABIO)  Caspase9 (#ET1603-27, rabbit monoclonal, HUABIO)  β-actin (#sc-47778, mouse monoclonal, Santa Cruz) | 1:1000  1:1000  1:1000  1:1000  1:1000  1:1000  1:1000  1:1000  1:5000 |

Table S4 Primer sequences for LAT1 siRNA

| Target gene | Primer | Primer sequences (5′–3′) |
| --- | --- | --- |
| siLAT1 001 | Forward  Reverse | GUCCCAUCAAGGUGAAUCUTT  AGAUUCACCUUGAUGGGACTT |
| siLAT1 002 | Forward  Reverse | CUACGGAGGAUGGAACUAUTT  AUAGUUCCAUCCUCCGUAGTT |
| siLAT1 003 | Forward  Reverse | CUGGUGUUCACGUGUAUCATT  UGAUACACGUGAACACCAGTT |
