# Supplementary material for: LAT1-mediated delivery of engineered R13A-MOTS-c attenuates radiation-induced lung injury via Nrf2 activation and mitochondrial protection
Source: Redox Biol. 2026 May 9;94:104204. doi: 10.1016/j.redox.2026.104204 (PMC13199819; doi:10.1016/j.redox.2026.104204)
Supplement: Multimedia component 2 [file mmc2.doc]

Product Name :MOTS-C

Analyst :YL

Lot No. :P230317-LR448144

Column :Kromasil C18,4.6*250mm,5um

Solvent A :0.1%Trifluoroacetic in 100% Acetonitrile

Solvent B :0.1%Trifluoroacetic in 100% Water

Gradient : A B

0.0min 27% 73%

25.0min 52% 48%

25.1min 100% 0%

30.0min Stop

Flow rate :1.0ml/min

Wavelength :220nm

Volume :20ul

────────────────────────

Rank Time Quantity Area Height

────────────────────────

1 8.375 0.2601 30059 2459

2 9.411 98.92 11430320 757947

3 9.753 0.1457 16838 2886

4 10.208 0.6786 78424 6411

────────────────────────

Total 100 11555641 769703
