# Supplementary material for: LAT1-mediated delivery of engineered R13A-MOTS-c attenuates radiation-induced lung injury via Nrf2 activation and mitochondrial protection
Source: Redox Biol. 2026 May 9;94:104204. doi: 10.1016/j.redox.2026.104204 (PMC13199819; doi:10.1016/j.redox.2026.104204)
Supplement: Multimedia component 4 [file mmc4.pptx]

## Slide 1
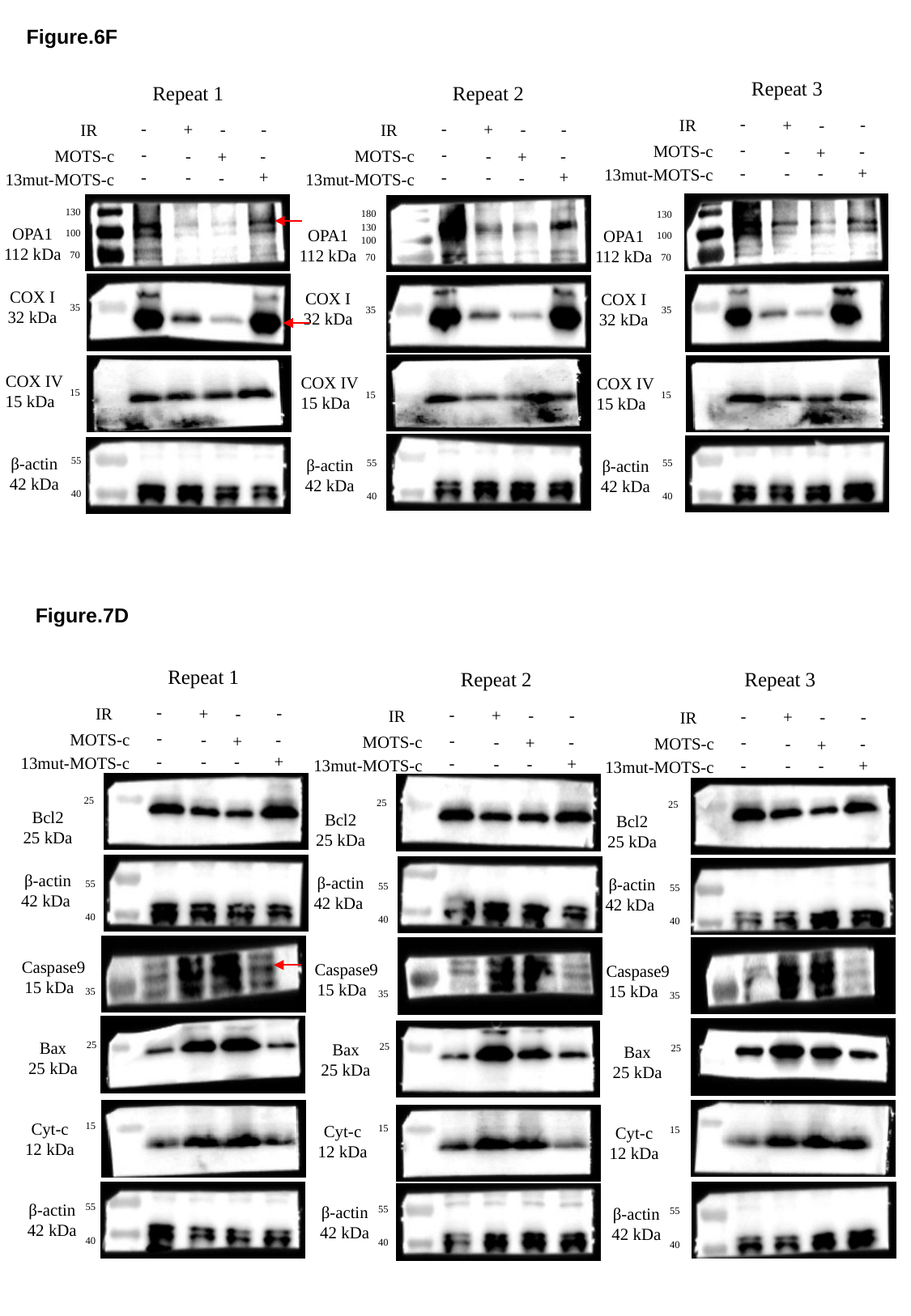

Figure.6F
Repeat 3
Repeat 1
Repeat 2
-
-
+
-
IR
-
-
-
-
+
-
+
-
IR
IR
-
MOTS-c
-
-
+
-
-
MOTS-c
-
-
MOTS-c
-
-
+
+
-
-
+
-
13mut-MOTS-c
-
-
-
+
-
+
-
-
13mut-MOTS-c
13mut-MOTS-c
130
180
130
130
OPA1
112 kDa
OPA1
112 kDa
OPA1
112 kDa
100
100
100
70
70
70
COX I
32 kDa
COX I
32 kDa
COX I
32 kDa
35
35
35
COX IV
15 kDa
COX IV
15 kDa
COX IV
15 kDa
15
15
15
β-actin
42 kDa
55
β-actin
42 kDa
β-actin
42 kDa
55
55
40
40
40
Figure.7D
Repeat 1
Repeat 2
Repeat 3
-
-
+
-
IR
-
-
+
-
IR
-
-
+
-
IR
-
MOTS-c
-
-
-
+
MOTS-c
-
-
-
+
MOTS-c
-
-
+
-
-
+
-
13mut-MOTS-c
-
-
+
-
-
-
+
13mut-MOTS-c
-
13mut-MOTS-c
25
25
25
Bcl2
25 kDa
Bcl2
25 kDa
Bcl2
25 kDa
β-actin
42 kDa
β-actin
42 kDa
β-actin
42 kDa
55
55
55
40
40
40
Caspase9
15 kDa
Caspase9
15 kDa
Caspase9
15 kDa
35
35
35
Bax
25 kDa
25
Bax
25 kDa
25
Bax
25 kDa
25
Cyt-c
12 kDa
15
Cyt-c
12 kDa
15
Cyt-c
12 kDa
15
β-actin
42 kDa
55
β-actin
42 kDa
55
β-actin
42 kDa
55
40
40
40

## Slide 2
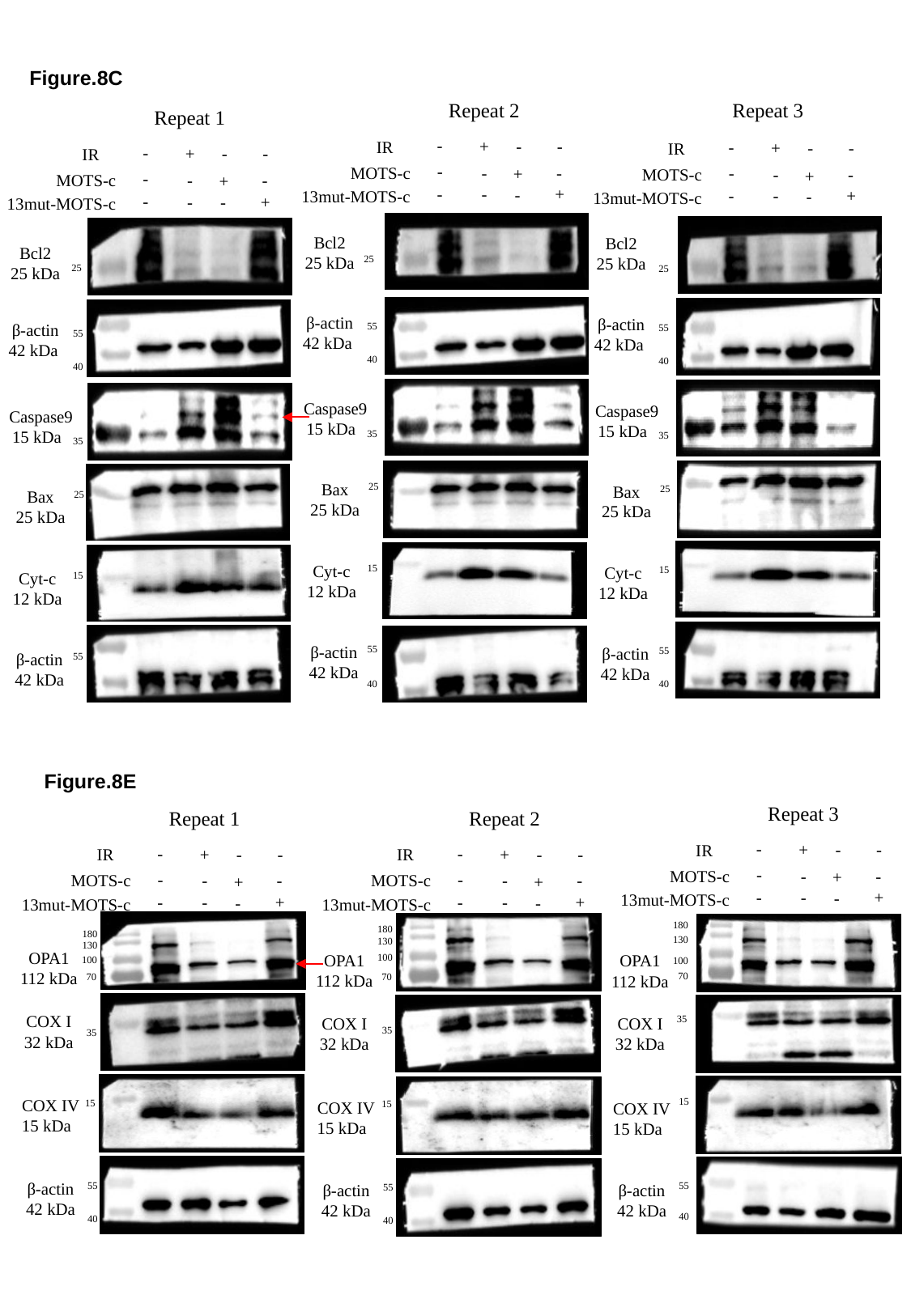

Figure.8C
Repeat 2
Repeat 3
Repeat 1
-
-
+
-
IR
-
-
+
-
IR
-
-
+
-
IR
-
MOTS-c
-
-
-
+
MOTS-c
-
-
+
-
MOTS-c
-
-
+
-
-
+
-
-
-
+
13mut-MOTS-c
-
13mut-MOTS-c
-
-
+
-
13mut-MOTS-c
Bcl2
25 kDa
Bcl2
25 kDa
Bcl2
25 kDa
25
25
25
β-actin
42 kDa
β-actin
42 kDa
β-actin
42 kDa
55
55
55
40
40
40
Caspase9
15 kDa
Caspase9
15 kDa
Caspase9
15 kDa
35
35
35
Bax
25 kDa
25
Bax
25 kDa
25
Bax
25 kDa
25
Cyt-c
12 kDa
15
Cyt-c
12 kDa
15
Cyt-c
12 kDa
15
β-actin
42 kDa
55
β-actin
42 kDa
55
β-actin
42 kDa
55
40
40
Figure.8E
Repeat 3
Repeat 1
Repeat 2
-
-
+
-
IR
-
-
-
-
+
-
+
-
IR
IR
-
MOTS-c
-
-
+
-
-
MOTS-c
-
-
MOTS-c
-
-
+
+
-
-
+
-
13mut-MOTS-c
-
-
-
+
-
+
-
-
13mut-MOTS-c
13mut-MOTS-c
180
180
180
130
130
130
OPA1
112 kDa
OPA1
112 kDa
OPA1
112 kDa
100
100
100
70
70
70
COX I
32 kDa
COX I
32 kDa
35
COX I
32 kDa
35
35
15
COX IV
15 kDa
15
COX IV
15 kDa
15
COX IV
15 kDa
β-actin
42 kDa
55
55
β-actin
42 kDa
β-actin
42 kDa
55
40
40
40

## Slide 3
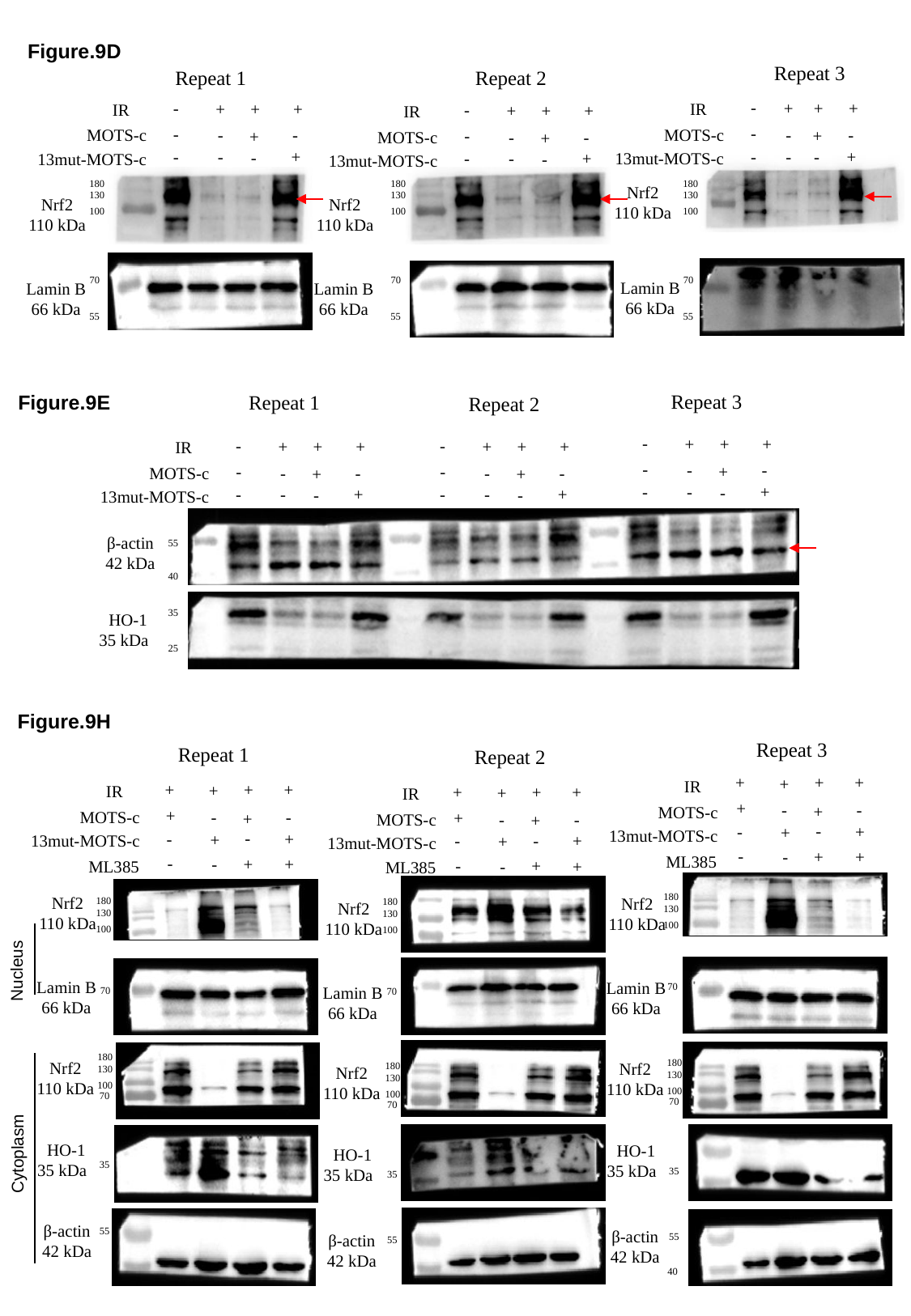

Figure.9D
Repeat 3
Repeat 1
Repeat 2
-
-
+
+
+
+
IR
+
+
IR
-
+
+
+
IR
-
-
MOTS-c
-
-
MOTS-c
-
-
-
+
+
MOTS-c
-
-
+
-
-
+
-
-
-
+
-
13mut-MOTS-c
-
-
+
13mut-MOTS-c
-
13mut-MOTS-c
180
180
180
Nrf2
110 kDa
130
130
130
Nrf2
110 kDa
Nrf2
110 kDa
100
100
100
70
70
70
Lamin B
66 kDa
Lamin B
66 kDa
Lamin B
66 kDa
55
55
55
Repeat 3
Repeat 1
Repeat 2
-
+
+
+
-
-
+
+
+
+
+
+
IR
-
-
-
-
-
+
MOTS-c
-
-
-
-
+
+
-
-
+
-
-
-
-
+
-
+
-
-
13mut-MOTS-c
β-actin
42 kDa
55
40
35
HO-1
35 kDa
25
Figure.9E
Figure.9H
Repeat 3
Repeat 1
Repeat 2
+
+
+
+
IR
+
+
+
+
IR
+
+
+
+
IR
+
-
-
+
MOTS-c
+
MOTS-c
-
-
+
+
MOTS-c
-
-
+
-
-
+
+
13mut-MOTS-c
-
-
+
+
-
13mut-MOTS-c
-
+
+
13mut-MOTS-c
-
+
-
+
ML385
-
+
-
+
-
+
ML385
-
+
ML385
180
Nrf2
110 kDa
Nrf2
110 kDa
180
180
Nrf2
110 kDa
130
130
130
100
100
100
Nucleus
Lamin B
66 kDa
Lamin B
66 kDa
70
Lamin B
66 kDa
70
70
180
180
Nrf2
110 kDa
Nrf2
110 kDa
180
Nrf2
110 kDa
130
130
130
100
100
100
70
70
70
HO-1
35 kDa
HO-1
35 kDa
Cytoplasm
HO-1
35 kDa
35
35
35
β-actin
42 kDa
55
β-actin
42 kDa
β-actin
42 kDa
55
55
40

## Slide 4
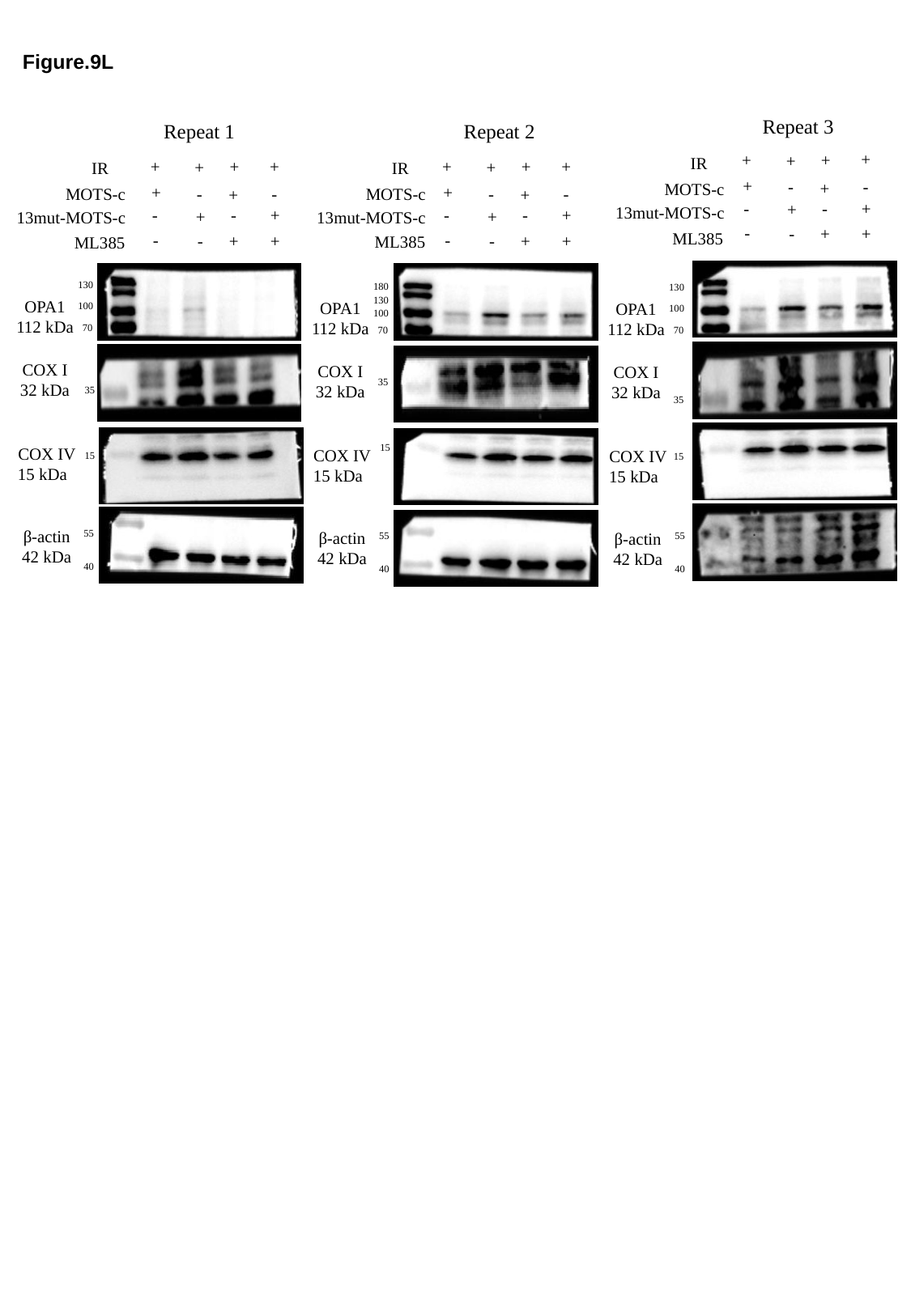

Figure.9L
Repeat 3
Repeat 1
Repeat 2
+
+
+
+
IR
+
+
+
+
+
+
+
+
IR
IR
+
-
-
+
MOTS-c
+
+
MOTS-c
-
-
MOTS-c
-
-
+
+
-
-
+
+
13mut-MOTS-c
-
-
-
-
+
+
+
+
13mut-MOTS-c
13mut-MOTS-c
-
+
-
+
ML385
-
-
+
+
-
+
-
+
ML385
ML385
130
180
130
130
OPA1
112 kDa
OPA1
112 kDa
OPA1
112 kDa
100
100
100
70
70
70
COX I
32 kDa
COX I
32 kDa
COX I
32 kDa
35
35
35
15
COX IV
15 kDa
COX IV
15 kDa
COX IV
15 kDa
15
15
β-actin
42 kDa
55
β-actin
42 kDa
β-actin
42 kDa
55
55
40
40
40

## Slide 5
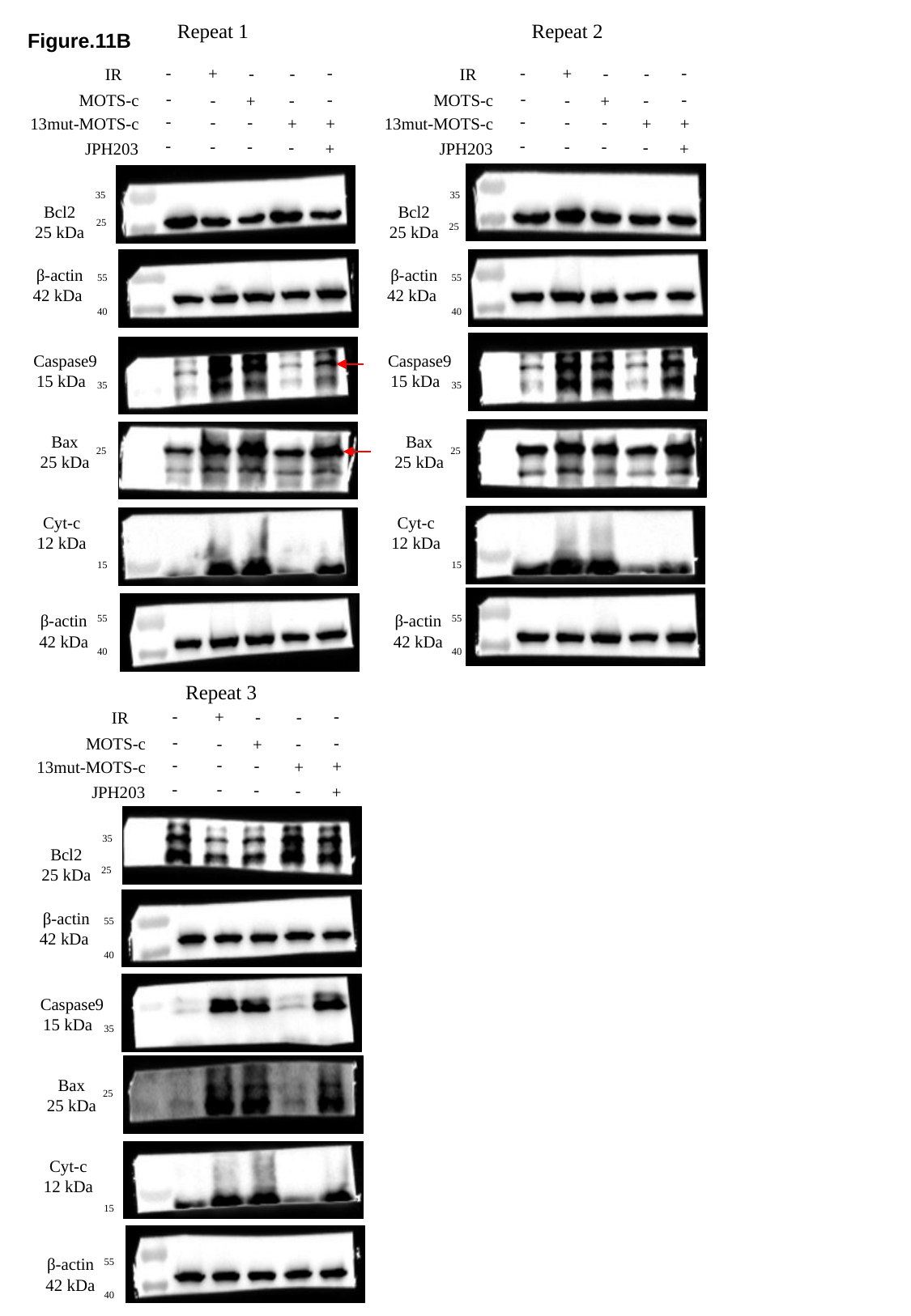

Repeat 1
Repeat 2
Figure.11B
-
-
-
-
-
-
+
-
+
-
IR
IR
-
-
-
-
MOTS-c
-
-
MOTS-c
-
-
+
+
-
-
-
-
-
-
+
+
13mut-MOTS-c
13mut-MOTS-c
+
+
-
-
-
-
-
-
-
-
JPH203
JPH203
+
+
35
35
Bcl2
25 kDa
Bcl2
25 kDa
25
25
β-actin
42 kDa
β-actin
42 kDa
55
55
40
40
Caspase9
15 kDa
Caspase9
15 kDa
35
35
Bax
25 kDa
Bax
25 kDa
25
25
Cyt-c
12 kDa
Cyt-c
12 kDa
15
15
β-actin
42 kDa
β-actin
42 kDa
55
55
40
40
Repeat 3
-
-
-
+
-
IR
-
-
MOTS-c
-
-
+
-
-
-
+
13mut-MOTS-c
+
-
-
-
-
JPH203
+
35
Bcl2
25 kDa
25
β-actin
42 kDa
55
40
Caspase9
15 kDa
35
Bax
25 kDa
25
Cyt-c
12 kDa
15
β-actin
42 kDa
55
40

## Slide 6
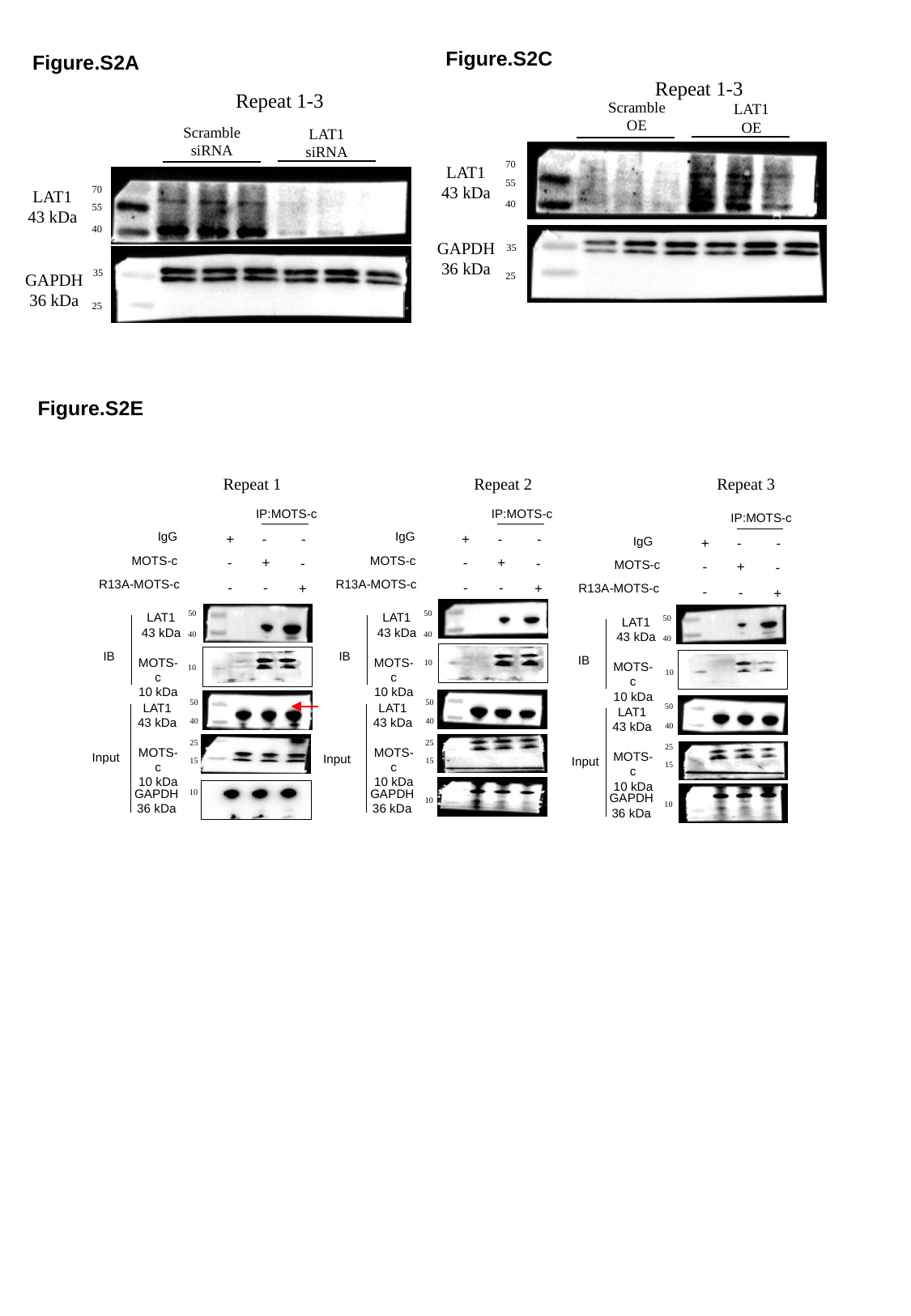

Figure.S2C
Figure.S2A
Repeat 1-3
Scramble
OE
LAT1
OE
70
LAT1
43 kDa
55
40
GAPDH
36 kDa
35
25
Repeat 1-3
Scramble
siRNA
LAT1
siRNA
70
LAT1
43 kDa
55
40
35
GAPDH
36 kDa
25
Figure.S2E
Repeat 1
Repeat 2
Repeat 3
IP:MOTS-c
IP:MOTS-c
IP:MOTS-c
IgG
IgG
-
-
-
-
+
+
IgG
-
-
+
MOTS-c
MOTS-c
-
-
+
+
-
-
MOTS-c
-
+
-
R13A-MOTS-c
R13A-MOTS-c
-
-
-
-
+
+
R13A-MOTS-c
-
-
+
LAT1
43 kDa
LAT1
43 kDa
50
50
LAT1
43 kDa
50
40
40
40
IB
IB
IB
MOTS-c
10 kDa
MOTS-c
10 kDa
10
MOTS-c
10 kDa
10
10
50
50
LAT1
43 kDa
LAT1
43 kDa
50
LAT1
43 kDa
40
40
40
25
25
25
MOTS-c
10 kDa
MOTS-c
10 kDa
MOTS-c
10 kDa
Input
Input
Input
15
15
15
GAPDH
36 kDa
GAPDH
36 kDa
10
GAPDH
36 kDa
10
10

## Slide 7
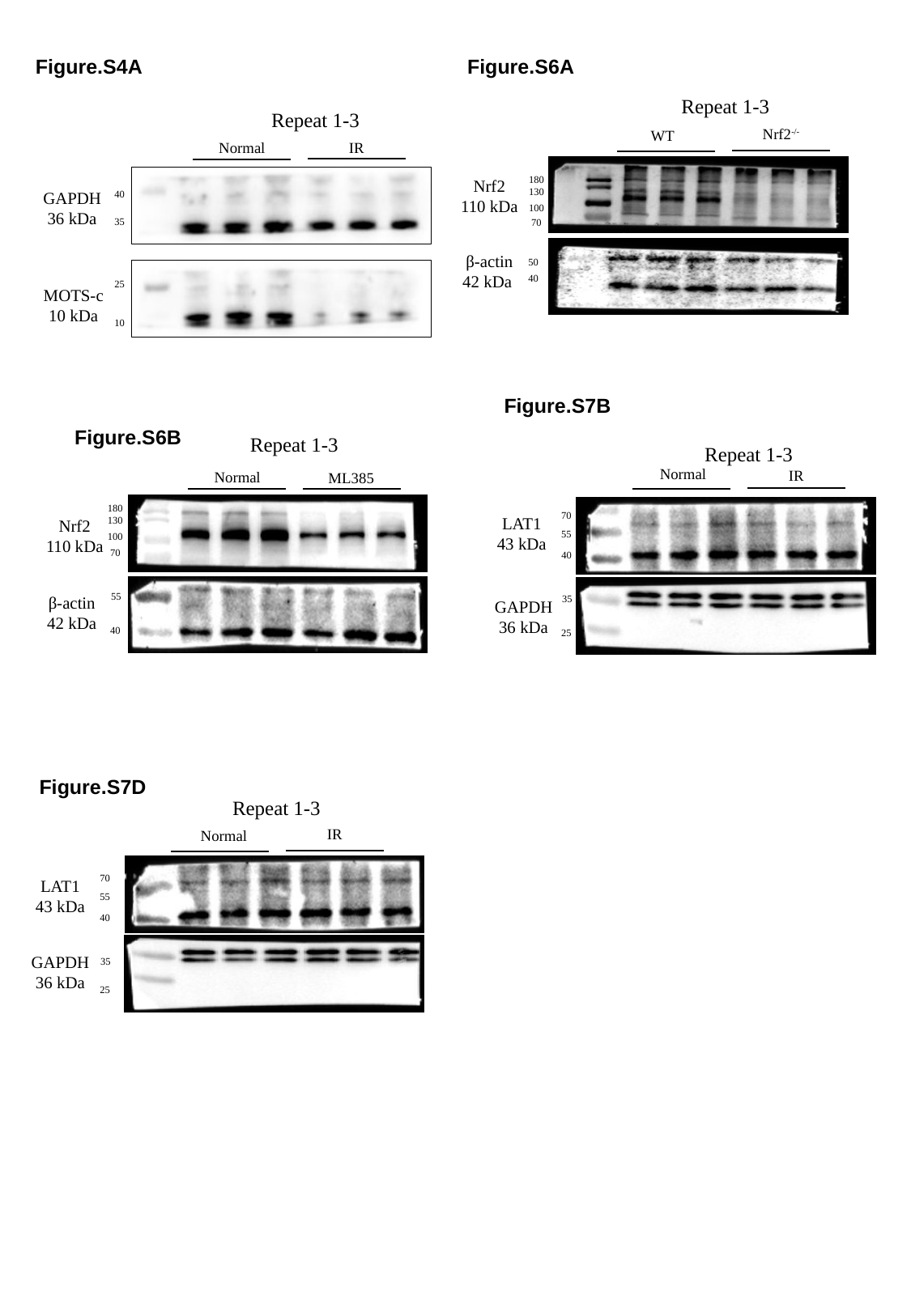

Figure.S6A
Figure.S4A
Repeat 1-3
Nrf2-/-
WT
180
Nrf2
110 kDa
130
100
70
β-actin
42 kDa
50
40
Repeat 1-3
Normal
IR
GAPDH36 kDa
40
35
25
MOTS-c
10 kDa
10
Figure.S7B
Figure.S6B
Repeat 1-3
Normal
ML385
180
130
Nrf2
110 kDa
100
70
55
β-actin
42 kDa
40
Repeat 1-3
Normal
IR
70
LAT1
43 kDa
55
40
35
GAPDH
36 kDa
25
Figure.S7D
Repeat 1-3
IR
Normal
70
LAT1
43 kDa
55
40
GAPDH
36 kDa
35
25

## Slide 8
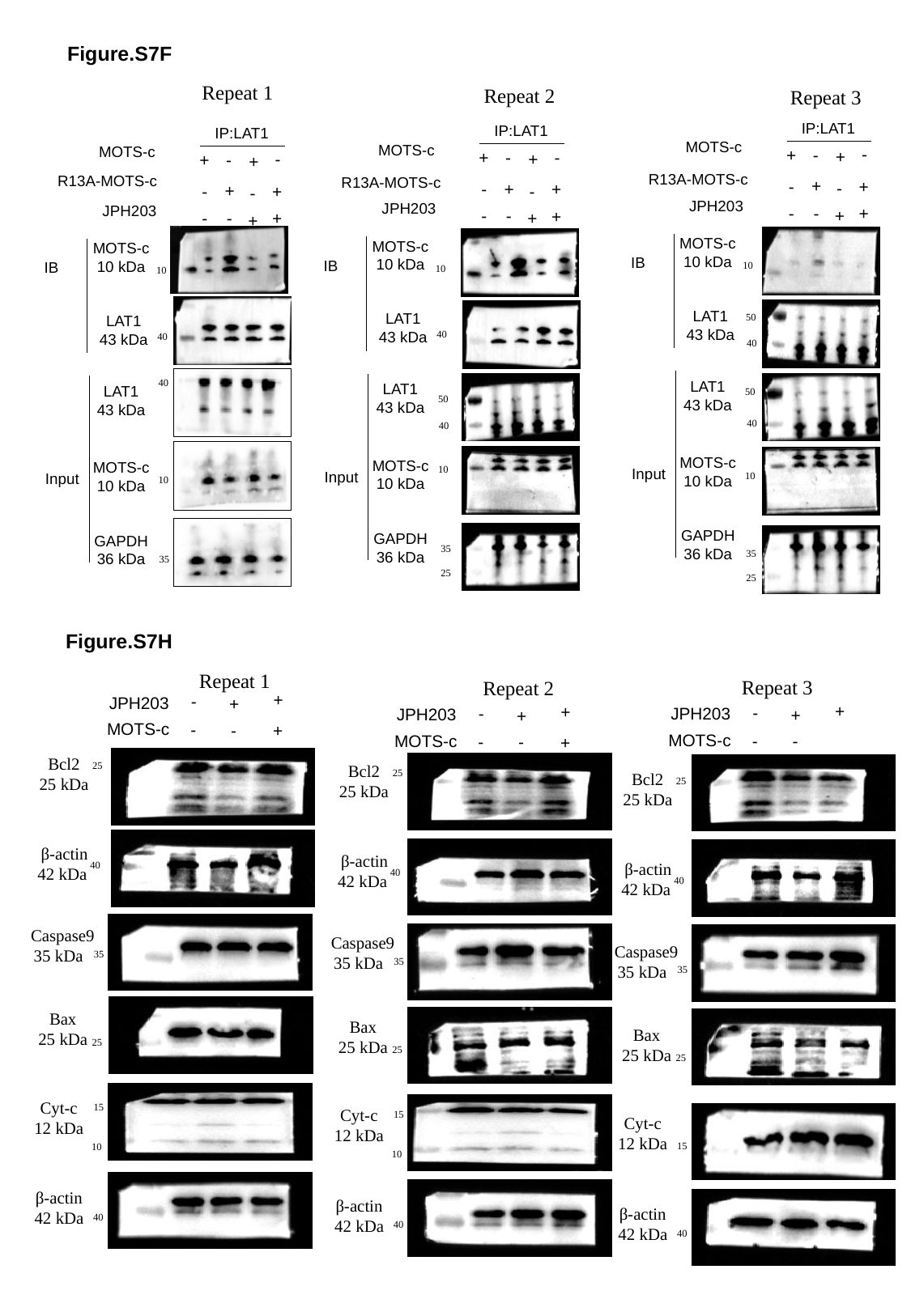

Figure.S7F
Repeat 1
IP:LAT1
MOTS-c
-
+
-
+
R13A-MOTS-c
+
-
+
-
JPH203
-
-
+
+
MOTS-c
10 kDa
IB
10
LAT1
43 kDa
40
40
LAT1
43 kDa
MOTS-c
10 kDa
Input
10
GAPDH
36 kDa
35
Repeat 2
IP:LAT1
MOTS-c
-
+
-
+
R13A-MOTS-c
+
-
+
-
JPH203
-
-
+
+
MOTS-c
10 kDa
IB
10
LAT1
43 kDa
40
LAT1
43 kDa
50
40
MOTS-c
10 kDa
10
Input
GAPDH
36 kDa
35
25
Repeat 3
IP:LAT1
MOTS-c
-
+
-
+
R13A-MOTS-c
+
-
+
-
JPH203
-
-
+
+
MOTS-c
10 kDa
IB
10
LAT1
43 kDa
50
40
LAT1
43 kDa
50
40
MOTS-c
10 kDa
Input
10
GAPDH
36 kDa
35
25
Figure.S7H
Repeat 1
Repeat 3
Repeat 2
+
-
JPH203
+
+
+
-
-
JPH203
JPH203
+
+
MOTS-c
-
-
+
MOTS-c
MOTS-c
-
-
-
-
+
Bcl2
25 kDa
25
Bcl2
25 kDa
25
Bcl2
25 kDa
25
β-actin
42 kDa
β-actin
42 kDa
β-actin
42 kDa
40
40
40
Caspase9
35 kDa
Caspase9
35 kDa
Caspase9
35 kDa
35
35
35
Bax
25 kDa
Bax
25 kDa
Bax
25 kDa
25
25
25
Cyt-c
12 kDa
15
Cyt-c
12 kDa
15
Cyt-c
12 kDa
15
10
10
β-actin
42 kDa
β-actin
42 kDa
β-actin
42 kDa
40
40
40
