# Supplementary material for: LAT1-mediated delivery of engineered R13A-MOTS-c attenuates radiation-induced lung injury via Nrf2 activation and mitochondrial protection
Source: Redox Biol. 2026 May 9;94:104204. doi: 10.1016/j.redox.2026.104204 (PMC13199819; doi:10.1016/j.redox.2026.104204)
Supplement: Multimedia component 5 [file mmc5.pptx]

## Slide 1
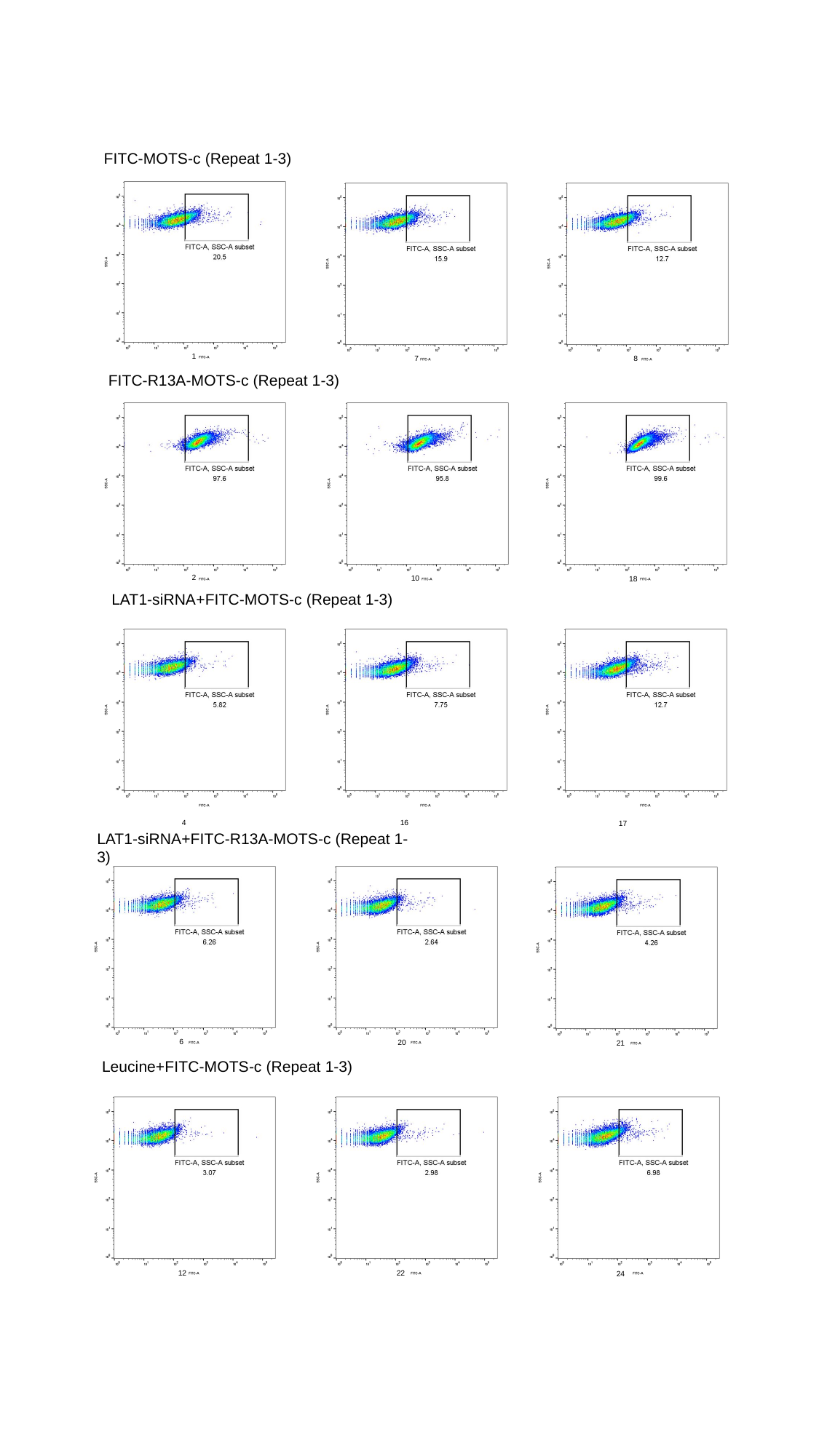

FITC-MOTS-c (Repeat 1-3)
1
7
8
FITC-R13A-MOTS-c (Repeat 1-3)
2
10
18
LAT1-siRNA+FITC-MOTS-c (Repeat 1-3)
4
16
17
LAT1-siRNA+FITC-R13A-MOTS-c (Repeat 1-3)
6
20
21
Leucine+FITC-MOTS-c (Repeat 1-3)
12
22
24

## Slide 2
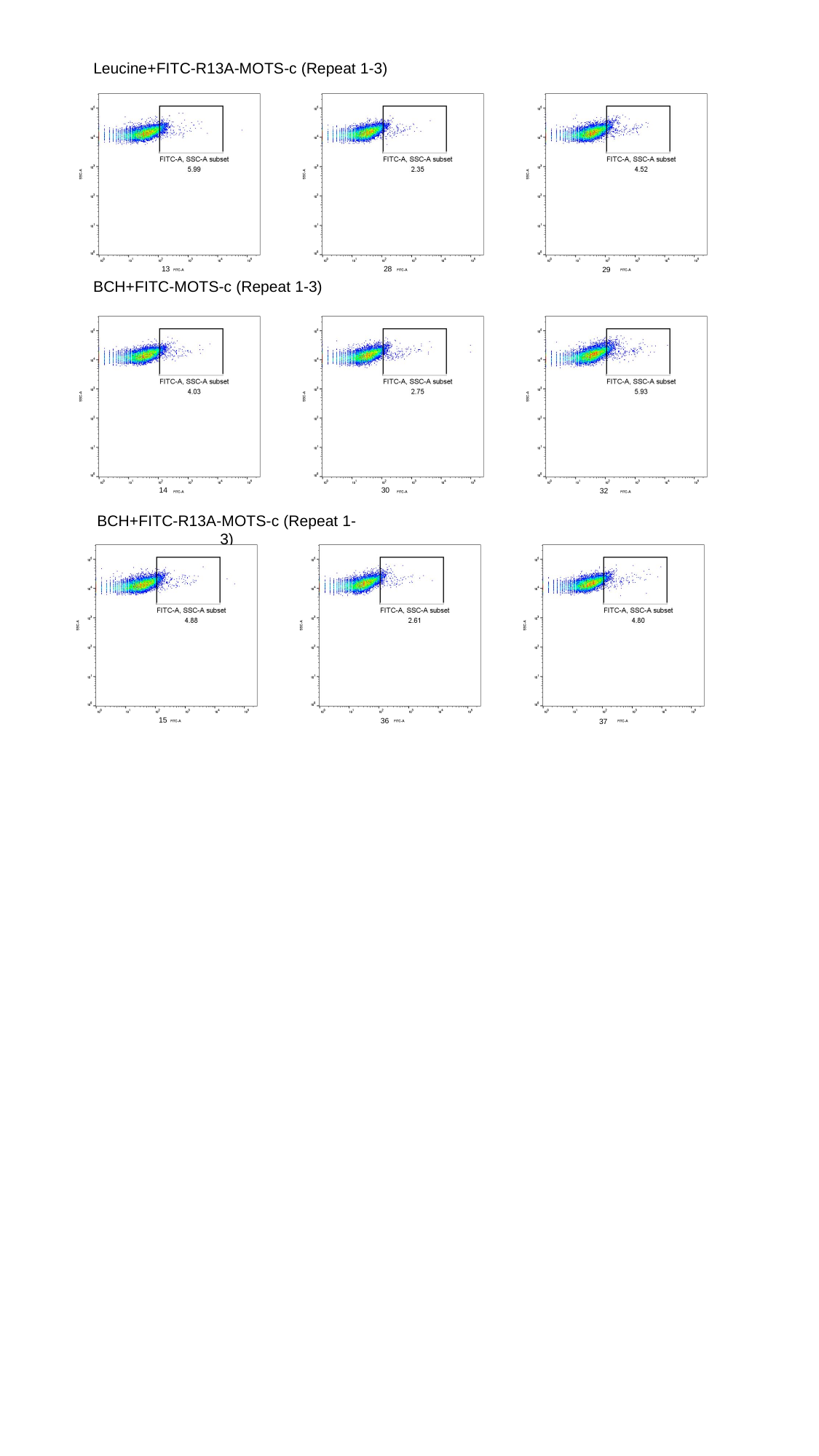

Leucine+FITC-R13A-MOTS-c (Repeat 1-3)
13
28
29
BCH+FITC-MOTS-c (Repeat 1-3)
14
30
32
BCH+FITC-R13A-MOTS-c (Repeat 1-3)
15
36
37
