# Supplementary material for: LAT1-mediated delivery of engineered R13A-MOTS-c attenuates radiation-induced lung injury via Nrf2 activation and mitochondrial protection
Source: Redox Biol. 2026 May 9;94:104204. doi: 10.1016/j.redox.2026.104204 (PMC13199819; doi:10.1016/j.redox.2026.104204)
Supplement: Multimedia component 6 [file mmc6.doc]

# Mass Spectrometry Report

| [M+2H]2+  [M+3H]3+  [M+4H]4+  [M+4H]4+  [M+5H]5+ | | | | | | | |
| --- | --- | --- | --- | --- | --- | --- | --- |
|  | Sample Description | | Instrument | SHIMADZU LCMS-2020 | | | |
|  | Analyzed date: | 2023/4/15 | Probe: | ESI | | Probe Bias: | ＋4.5kv |
|  | Analyst: | Shen | Nebulizer Gas Flow: | 1.5L/min | | Detector: | 1.2kv |
|  | Sample: | MR-16 | CDL: | -20.0v | | T. Flow: | 0.2ml/min |
|  | M.W.: | 2174.59 | CDL Temp.: | 250 | oC | B. Conc.: | 50%H2O/50%ACN |
|  | Lot. No.: | P230317-LR448144 | Block Temp.: | 400 | oC |  |  |
