# Supplementary figures and images for: LAT1-mediated delivery of engineered R13A-MOTS-c attenuates radiation-induced lung injury via Nrf2 activation and mitochondrial protection
Source: Redox Biol. 2026 May 9;94:104204. doi: 10.1016/j.redox.2026.104204 (PMC13199819; doi:10.1016/j.redox.2026.104204)

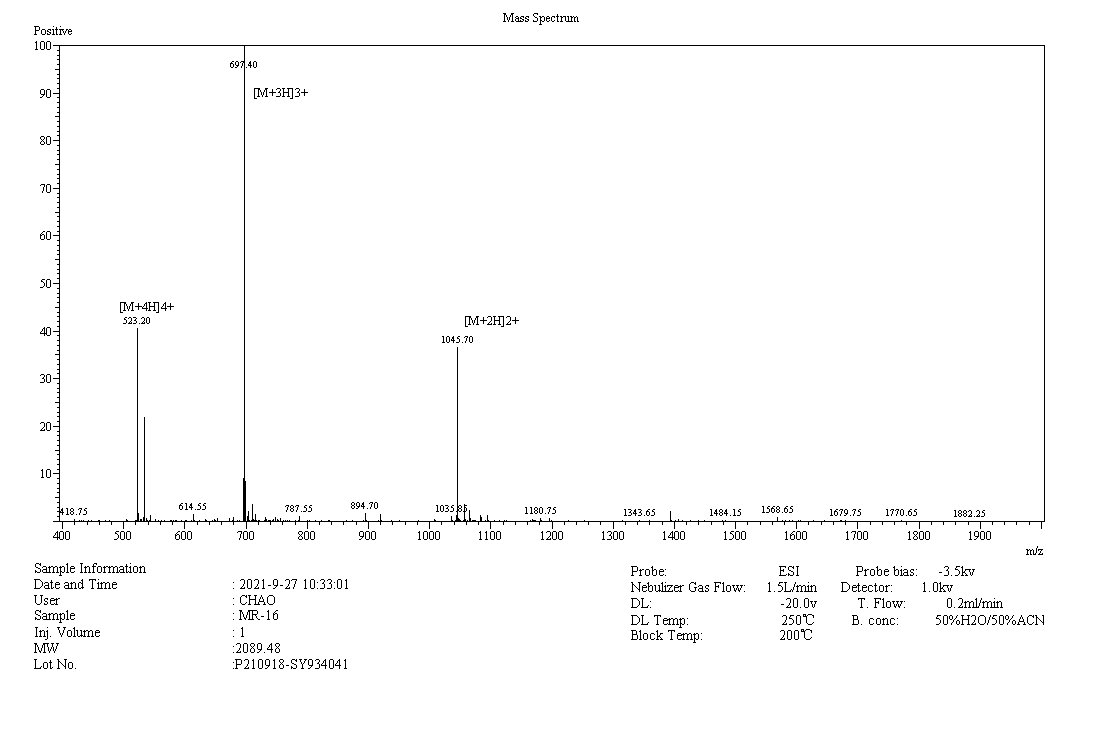

Supplement: Multimedia component 7 [file mmc7.doc]
